# Bactericidal activity of mammalian histones is caused by large membrane pore formation

**DOI:** 10.1016/j.celrep.2025.115658

**Published:** 2025-05-06

**Authors:** Leora Duong, Yonghan Wu, Summer J. Kasallis, Serena Abbondante, Paul J. Hurst, Michaela E. Marshall, Katherine McCarthy, Babu J.N. Reddy, Jean-Louis Bru, Kumar Perinbam, Eric Pearlman, Joseph P. Patterson, Steven P. Gross, Albert Siryaporn

**Affiliations:** 1 Department of Molecular Biology and Biochemistry, University of California, Irvine, Irvine, CA 92697, USA; 2 Department of Physics and Astronomy, University of California, Irvine, Irvine, CA 92697, USA; 3 Department of Ophthalmology, University of California, Irvine, Irvine, CA 92697, USA; 4 Department of Physiology and Biophysics, University of California, Irvine, Irvine, CA 92697, USA; 5 Department of Chemistry, University of California, Irvine, Irvine, CA 92697, USA; 6 Department of Developmental and Cell Biology, University of California, Irvine, Irvine, CA 92697, USA; 7 School of Basic and Applied Sciences, Dayananda Sagar University, Bengaluru, Karnataka 560078, India; 8 Lead contact

## Abstract

Histones have an important role in eukaryotic innate immunity, wherein histones co-localize with antimicrobial peptides (AMPs). The mechanism of histone cooperation with AMPs and the extent to which histones form pores both remain a mystery. Here, we show that histones form large pores in bacterial membranes that lack lipopolysaccharide (LPS) and that their antimicrobial effect is significantly stronger than that of the clinical AMP polymyxin B. We find that histones and AMPs together produce potent antimicrobial synergy through the formation of 26 nm pores, whereby the pore-forming activity of AMPs on LPS-containing membranes enables histones to enter the periplasmic space and subsequently attack unprotected membranes to create pores. We provide a mechanistic explanation for the long-standing observations of histone antimicrobial activity and demonstrate how antimicrobial synergy arises. The ubiquity of histones and AMPs in innate immunity has significant implications for organismal defense and can be leveraged for novel antibiotic strategies.

## In brief

Antibiotic resistance is an increasing global concern. Duong et al. uncover a synergistic mechanism between histones and antimicrobial peptides that could pave the way to novel antibacterial treatment strategies. These molecules together form large pores in bacterial membranes that are ultimately lethal to pathogenic bacteria.

## Graphical abstract

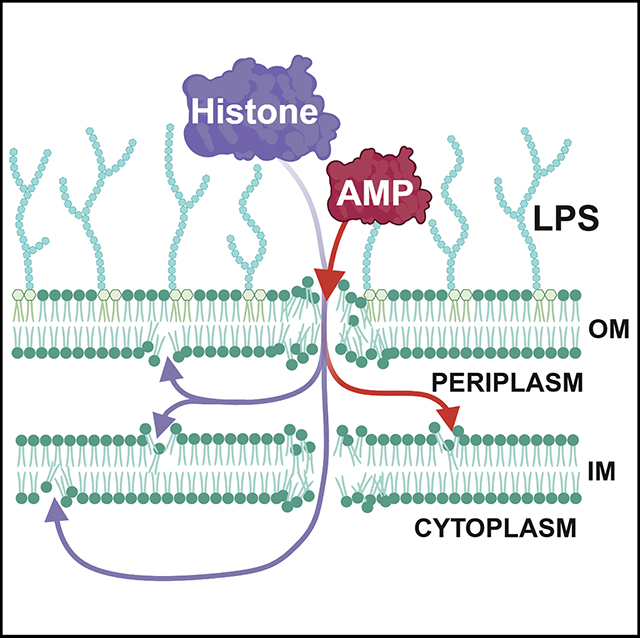

## INTRODUCTION

Histones are eukaryotic proteins that were originally reported as antimicrobial agents over 80 years ago^1^ but more recently have been studied mostly for their role in condensing chromosomal DNA and regulating transcription. Despite a lack of mechanistic understanding, the antimicrobial activity of histones has been implicated in multiple processes in mammalian innate immunity, particularly in neutrophil extracellular traps (NETs) and lipid droplets,^2–4^ where histones co-localize with antimicrobial peptides (AMPs) including LL-37 (cathelicidin). Since histones appear relatively inactive at physiological ionic concentrations,^5–7^ it has been suggested that they act as “helpers” to LL-37 and other AMPs. The combination of histones and LL-37 facilitates the uptake of both molecules into the bacterial cytoplasm, resulting in a positive feedback loop that synergistically increases the antibacterial effect of these molecules.^6^ However, the mechanism(s) responsible for histone and AMP uptake and how these molecules work together to increase antimicrobial activity is poorly understood.

AMPs including human LL-37 and the bacteria-derived polymyxins B (PMB) and E (colistin) form pores in Gram-negative bacteria by targeting the outer membrane (OM).^8,9^ LL-37 forms toroidal pores in membranes, whereas PMB and colistin target lipopolysaccharides (LPS) and rearrange them into rigid crystalline structures.^10–14^ Histone H2A, one of the most abundant histone proteins in lipid droplets,^15^ significantly increases the cytoplasmic uptake of LL-37, but it is unclear how H2A achieves this effect. A key challenge to understanding the antimicrobial activity of histones and AMPs is the lack of direct visualization of pores formed by either, potentially because AMP-induced pores may be transient or unstable.

The antimicrobial effect of histones is confusing because while histones enhance LL-37 uptake, histones alone appear unable to initiate the formation of membrane pores. The latter interpretation is supported by the observation that histones have minimal antimicrobial activity in physiologically-relevant conditions^5–7^ but have significant antimicrobial activity in low ionic conditions that destabilize the bacterial OM.^2,3,5,15–26^ If histones do not form membrane pores on their own, it is unclear how they translocate into the cytoplasm. Furthermore, even if histones entered the periplasm through a disrupted OM, it is unknown how histones could traverse the inner membrane (IM) into the cytoplasm given their apparent lack of membrane-permeabilizing activity.

We investigated the pore-forming activity of histone H2A toward *Pseudomonas aeruginosa* and *Escherichia coli*. *P. aeruginosa* is a Gram-negative opportunistic pathogen that causes a broad range of illnesses, including pneumonia and illnesses commonly associated with hospital-acquired infections.^27,28^ This clinically relevant pathogen is a major cause of severe lung infections in individuals with cystic fibrosis and of blinding corneal infections. A key challenge to clearing *P. aeruginosa* infections is the prevalence of antibiotic-resistant strains, which have been on the rise due to the overuse of antibiotics.^29,30^ While synergistic antimicrobial activity of histone H2A and LL-37 has been observed,^6^ we focus here on the combination of H2A with PMB due to the latter’s widespread use in healthcare.

We show that H2A alone has strong pore-forming activity toward all bacterial membrane surfaces except the OM outer leaflet, where H2A’s membrane-permeabilizing activity was inhibited by LPS. We find that PMB alone formed pores that were transient and were <5 nm, but in combination with H2A, they formed stable 26 nm pores in both the IM and OM. Our results suggest that the large pores created by the AMP+H2A combination arises from PMB providing the histones access to membranes that lack LPS, with subsequent large-pore generation by the histones on the unprotected membranes. The synergy between histones and PMB thus arises via orthogonal activity toward membranes. Comparable pore-forming effects were observed when H2A was combined with the innate immunity AMP LL-37 in place of PMB, which suggests that innate immunity through LL-37 and histones arises due to similar orthogonal activity.

## RESULTS

### Histone H2A potentiates PMB antimicrobial activity *in vitro* and *in vivo*

**Figure 1.**
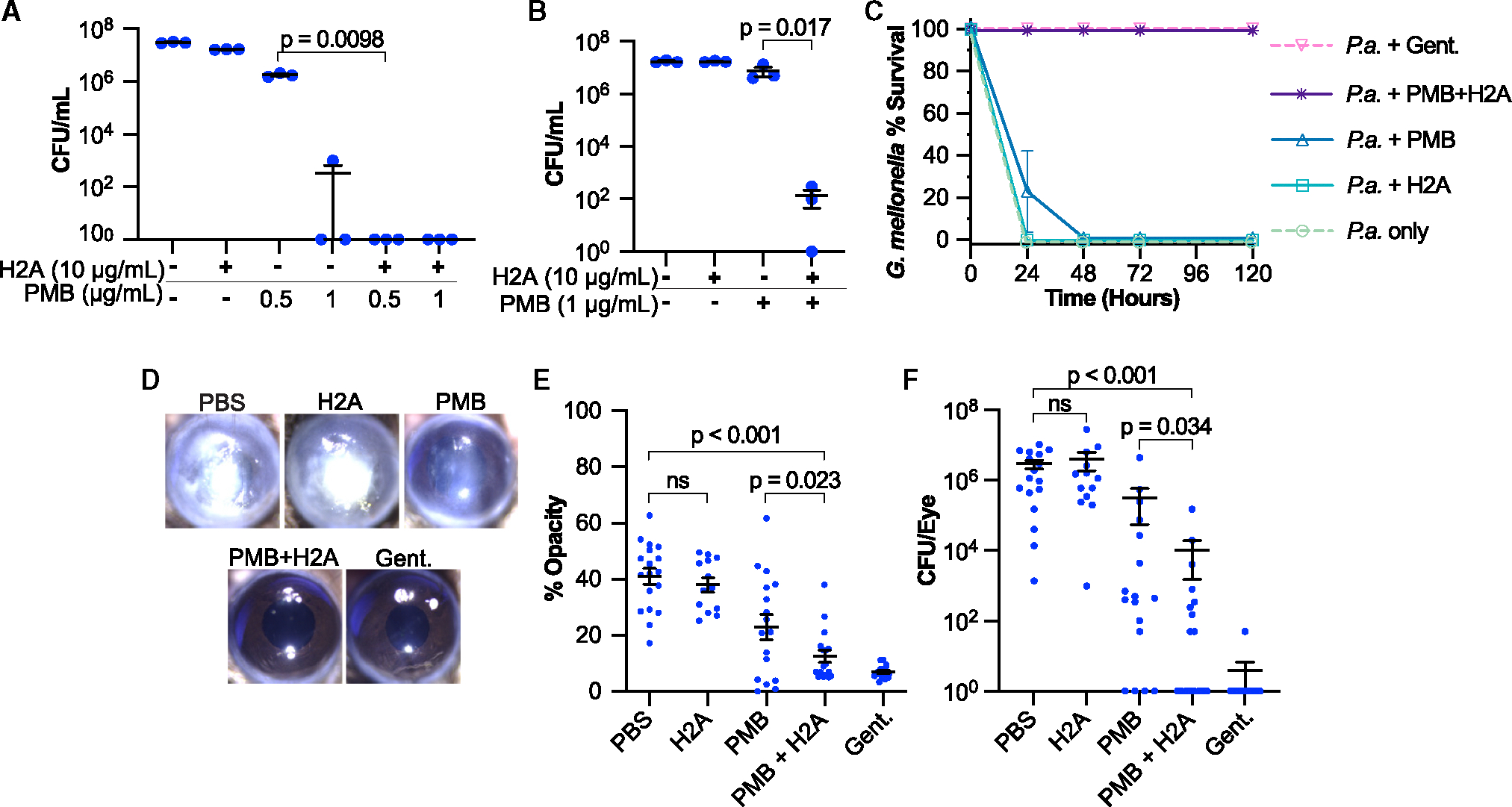
Effects of the PMB+H2A combination on bacterial growth and infection (A and B) Colony-forming units (CFUs) of (A) *E. coli* or (B) *P. aeruginosa* that were treated for 1 h with H2A, PMB, both, or untreated, and cultured on non-selective LB agar for 18 h. Data points represent independent experiments. (C) Survival of wax moth larvae (*G. mellonella*) infected with *P. aeruginosa* (P.a.) and treated with 10 μg/mL H2A, 1 μg/mL PMB, both H2A and PMB, 480 μg/mL gentamicin (Gent.), or untreated (*n* = 9 for each condition). (D–F) (D) Representative bright-field images, (E) corneal opacities, and (F) CFUs of mouse corneas 48 h after infection with *P. aeruginosa* and treatment with PBS, 10 μg H2A, 500 ng PMB, both H2A and PMB, or 30 μg gentamicin. Additional replicates of cornea images are in Figure S2B. Data points represent single mouse corneas. In plots, black bars indicate mean and error bars indicate standard error of the mean (SEM). For (A) and (B), *p* values were determined using two-tailed t tests with unequal variances. For (E) and (F), one-tailed t tests were performed. *p* >0.05 are denoted as non-significant (ns). See also Figures S1 and S2.

To determine the antimicrobial activity of H2A *in vitro*, we incubated *E. coli* for 1 h at 37°C with sub-minimal inhibitory concentrations (MICs) of either 0.5 μg/mL PMB (3.2 μM),^31^ 10 μg/mL histone H2A (0.7 μM, which is lower than the concentration found in human blood plasma following *E. coli* infection^32^), or both, and plated the bacteria on non-selective media. While the individual treatments of PMB or H2A alone only modestly decreased colony-forming units (CFUs), the combined PMB+H2A treatment reduced CFUs by over seven orders of magnitude (Figure 1A). The assessment of CFUs using non-selective plates suggests that the combined PMB+H2A treatment during the 1-h incubation is bactericidal. Tracking the growth in liquid cultures over the course of 24 h, PMB remained sub-inhibitory at a slightly higher concentration of 1 μg/mL, but the PMB+H2A combination completely inhibited growth (Figure S1A). The antimicrobial effect of PMB+H2A was more potent than the bactericidal aminoglycosides kanamycin and gentamicin, as well as the broad-spectrum protein synthesis inhibitor tetracycline at their respective standard-reported MICs^33^ (Figure S1B). To assess synergy, we performed checkerboard assays and computed a fractional inhibitory concentration (FIC) index, for which a value ≤0.5 is considered synergistic.^34^ The FIC of PMB+H2A was 0.16 (Figure S1C), indicating that this combination is strongly synergistic.

We hypothesized that the PMB+H2A dual treatment would be broadly effective against Gram-negative bacteria and thus assessed its efficacy on *P. aeruginosa*. The same concentrations of PMB and H2A used for *E. coli* assays were used against *P. aeruginosa* to determine relative antimicrobial efficacy. Similar to observations in *E. coli*, individual treatments of PMB or H2A alone had no significant impact on *P. aeruginosa* CFUs (Figure 1B). However, the combination of PMB+H2A reduced *P. aeruginosa* growth by more than five orders of magnitude on non-selective plates, indicating that the mechanism is bactericidal. In liquid cultures, growth was suppressed for 15 h (Figure S1D). Checkerboard assays revealed an FIC index of 0.19 (Figure S1E), indicating strong synergy.^34^ Kanamycin, gentamicin, and tetracycline at the same concentrations as used against *E. coli* were ineffective at inhibiting *P. aeruginosa* growth (Figure S1F). PMB+H2A also completely inhibited the growth of a cystic fibrosis clinical isolate of *P. aeruginosa* (Figure S1G). These data indicate that the PMB +H2A combination is likely synergistic and bactericidal for multiple Gram-negative bacteria in addition to *P. aeruginosa* and *E. coli*.

To assess the PMB+H2A antimicrobial activity *in vivo*, we began with wax moth larvae (*Galleria mellonella*).^35,36^ *P. aeruginosa* was mixed with either PMB, H2A, or both, at the same concentrations as used in the *in vitro* experiments, and immediately injected into larvae. No larvae injected with phosphate-buffered saline (PBS) or H2A alone survived after 24 h (Figure 1C). PMB treatment alone increased the survival of 22% of the larvae up to 24 h post-infection. Remarkably, all larvae treated with PMB+H2A survived for at least 5 days, comparable to the group treated with gentamicin (Figure 1C).

The PMB+H2A combination was next assessed in a murine model of corneal infection using *P. aeruginosa*. The corneal epithelium was abraded and 5 × 10^4^ *P. aeruginosa* strain PAO1 cells were added topically as described.^37^ Following infection, mice were given topical PMB, H2A, PMB+H2A, or gentamicin three times per day for 2 days (Figure S2A). We used the lowest possible PMB dose that caused a statistically significant reduction in CFUs; H2A concentration was the same molar ratio as used with PMB *in vitro*. Total corneal disease was quantified using image analysis because the bacterial growth and infiltration of neutrophils leads to increased corneal opacity.^38,39^ We performed image analysis of corneal opacity 48 h post-infection, and homogenized corneas were plated in CFU assays to quantify viable bacteria.

Consistent with previous reports,^27^ *P. aeruginosa* infection resulted in severe corneal opacity, while corneas of gentamicin-treated mice remained clear (Figures 1D, 1E, and S2B). Corneal opacity of H2A-treated or PMB-treated mice was not significantly different from untreated (PBS) mice. In marked contrast, corneas that received PMB+H2A together showed less corneal disease, significantly lower than PMB alone (Figures 1D, 1E, and S2B). Significant bacterial reduction was observed from PMB+H2A treatments compared to H2A or PMB alone (Figure 1F). In particular, the effects on corneal opacity and CFU counts resembled mice that were given gentamicin (Figures 1E and 1F). Thus, the combined PMB+H2A treatment was effective in two *in vivo* models of *P. aeruginosa* infection, demonstrating significant synergy. We devoted the remainder of our study to understanding the basis of this AMP+H2A synergy and addressing outstanding mechanistic questions about histone activity.

### The combination of H2A with PMB causes pore formation in the outer and inner membranes of bacteria

**Figure 2.**
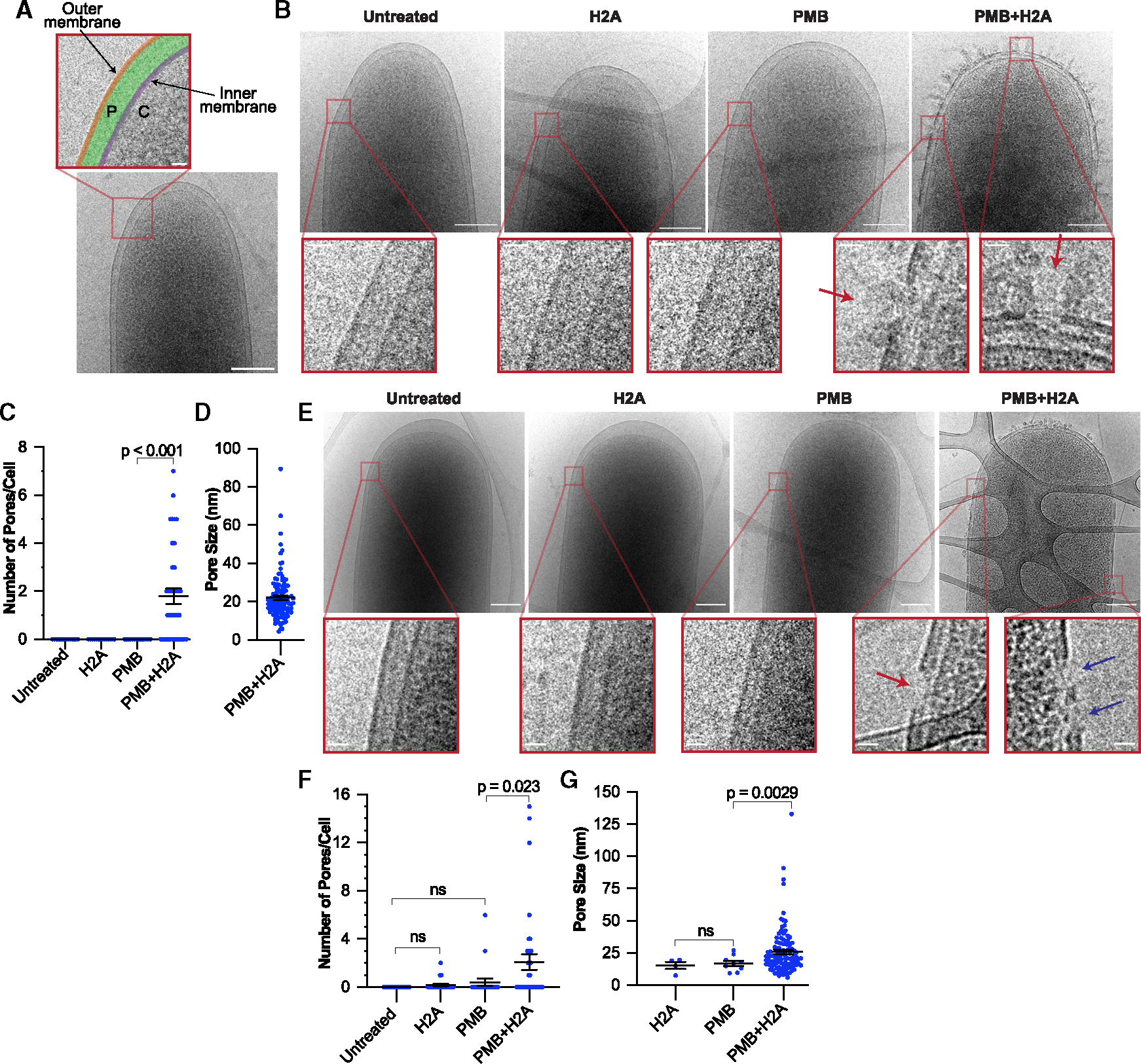
Visualization of *P. aeruginosa* and *E. coli* pores (A) Cryo-EM image of untreated *P. aeruginosa*, false colored to indicate the inner membrane (purple), outer membrane (orange), periplasm (P, green), and cytoplasm (C). (B) Representative cryo-EM images of *P. aeruginosa* cells that were treated for 30 min with 10 μg/mL H2A, 1 μg/mL PMB, both H2A and PMB, or untreated. Red arrows point to pores in the OM. (C and D) (C) Number of OM pores per cell and (D) OM pore sizes for the full set of cryo-EM data for *P. aeruginosa*. Cells lacking pores excluded in (D). (E) Representative cryo-EM images of *E. coli* cells that were treated for 15 min with 10 μg/mL H2A, 1 μg/mL PMB, both H2A and PMB, or untreated. Red arrow points to an OM pore and blue arrows point to pores that span both the OM and IM. In (A), (B), and (E), scale bars represent 200 nm in main images and 25 nm in magnified images. (F and G) (F) Number of OM pores per cell and (G) OM pore sizes for the full set of cryo-EM data for *E. coli*. Cells lacking pores excluded in (G). Data points represent individual bacterial cells in (C) and (F) and individual pores in (D) and (G). In all plots, black bars represent the means and error bars indicate SEMs. *p* values indicated were obtained using two-tailed t tests with unequal variances, and *p* > 0.05 are denoted as non-significant (ns). See also Figures S2 and S3.

The strong antimicrobial activity observed using PMB+H2A could be due to (1) the formation of a greater number of pores than with PMB or H2A alone, (2) larger pores than with PMB or H2A alone, or (3) that the pores created by PMB+H2A are more stable. We visualized bacterial membranes using cryogenic transmission electron microscopy (cryoEM), which preserves membrane structures and enables visualization of both the OM and IM (Figure 2A). *P. aeruginosa* were treated for only 30 min to capture potential early-stage membrane permeabilization events and due to significant lysis of cells by the PMB+H2A combined treatment beyond this time.

The combined PMB+H2A treatment caused significant disruptions of the OM (Figures 2A and S2C), which we refer to here as pores. Up to 7 pores per cell were detected in the OM, with pores averaging 22.1 nm in diameter (Figures 2B–2D). No pores or obvious deformations of either membrane were observed in untreated cells or cells treated with H2A or PMB alone (Figures 2B–2D). Additional pore analysis was performed using *E. coli*. Similar to the observations in *P. aeruginosa*, *E. coli* cells treated with PMB+H2A contained multiple OM and IM pores per cell. Up to 15 pores that averaged 25.8 nm in diameter were observed in the OM (Figures 2E–2G and S2C). No pores were detected in untreated bacteria, and OM pores were detected in 10% of bacteria that were treated with H2A or PMB alone (Figures 2F, 2G, and S2C). In the rare cases that OM pores were observed, they were 15.4 and 17 nm for H2A or PMB treatment, respectively (Figures 2F and 2G). A total of 58% of PMB+H2A-treated cells did not contain pores, which we attribute to the brief treatment duration. The lack of pores or obvious membrane deformations following individual PMB treatments could be attributed to the possibility that pores formed by PMB are transient or are below our size-resolution limit (<5 nm).

Pores were also identified and characterized using regular transmission electron microscopy (TEM). No pores were observed in *E. coli* treated with H2A or PMB alone, whereas PMB+H2A-induced deformations were typically round and concentrated near cell poles, consistent with pore formation (Figure S3A). The average diameter of the round membrane deformations was 17.1 nm (Figure S3B). Similarly, high-contrast membrane deformations were observed for *P. aeruginosa* treated with the PMB+H2A combination, but not in individually treated bacteria (Figure S3C). Together, the cryoEM and TEM data demonstrate that the combination of PMB+H2A causes pore formation in both the OM and IM of *P. aeruginosa* and *E. coli*.

### Pores increase molecular transport and H2A localization

If the OM and IM pores caused by PMB+H2A are functional and increase membrane permeability, we would expect cells to uptake extracellular molecules into the cytoplasm. We used propidium iodide (PI), fluorescently labeled dextran, and fluorescently labeled histone H2A to test OM and IM permeability. PI is a small molecule (668 g/mol) that does not cross intact membranes. Treatment using PMB alone across a range of sub-MICs significantly increased cytoplasmic PI fluorescence in *E. coli* (Figures S4A–S4D), indicating that PMB alone increases both OM and IM membrane permeability. H2A alone had no impact on fluorescence, consistent with no change in IM permeability. The PMB+H2A combination increased PI fluorescence by 16-fold compared to PMB treatment alone (Figure S4B), indicating that H2A significantly increases PMB-induced membrane permeabilization. This underscores the magnitude of the PMB +H2A synergy.

**Figure 3.**
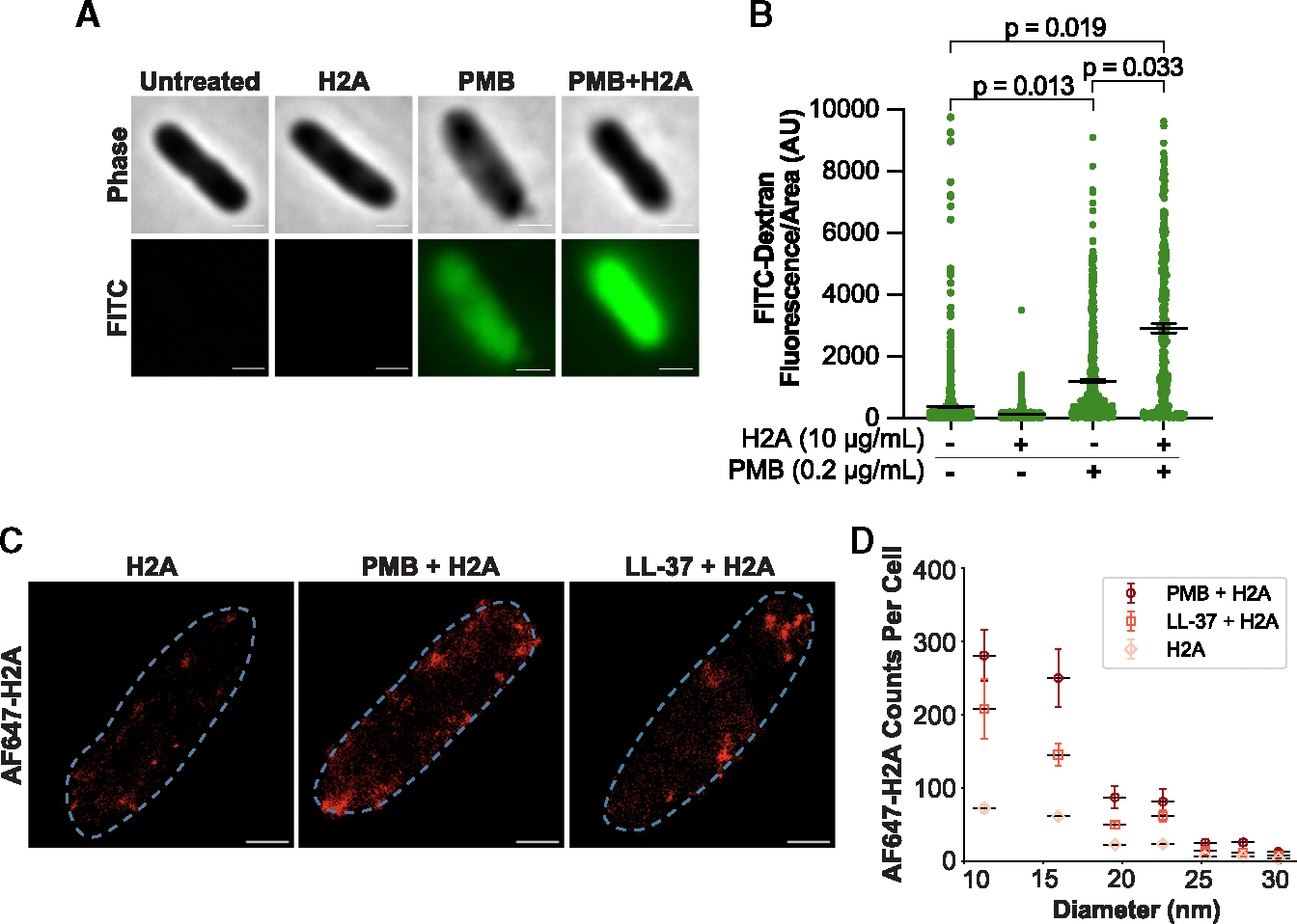
Assessment of membrane permeability and histone localization (A) Representative phase-contrast and FITC fluorescence images of *E. coli* incubated with 17 nm FITC-dextran following treatments of 10 μg/mL H2A, 0.2 μg/mL PMB, or both. (B) Mean intracellular FITC fluorescence for the full set of data represented in (A). A minor subset of points above the vertical axis maximum are not displayed; full data are available in Data S1. Scale bars represent 1 μm, and *p* values were obtained using two-tailed t tests with unequal variances. (C) Representative dSTORM images of *E. coli* treated with 10 μg/mL fluorescent H2A (AF647-H2A) alone or in combination with either 1 μg/mL PMB or 20 μg/mL LL-37. Scale bars represent 500 nm. (D) Distribution of AF647-H2A counts per cell for the full set of dSTORM data represented in (A). Counts are from multiple cluster sizes. Counts for clusters >30 nm are not displayed. Data points in (B) and (D) represent individual cells from three independent experiments. Black bars represent mean and error bars indicate SEM. See also Figures S4–S7.

We tracked fluorescent 150 kDa dextran (fluorescein isothiocyanate [FITC]-dextran), which is approximately 17 nm in diameter (see STAR Methods), to assess whether the pores allow the entry of large molecules into the cytoplasm. FITC-dextran fluorescence was not detected in untreated and H2A-treated cells (Figures 3A and 3B), consistent with the interpretation that H2A does not induce membrane pores in the OM. PMB alone increased FITC-dextran uptake by 3-fold, whereas PMB+H2A increased FITC-dextran uptake 8-fold (Figures 3A and 3B). This result supports our finding that PMB creates pores that are at least 17 nm in diameter and that the PMB+H2A combination significantly increases the size and number of pores.

To further characterize the activity of PMB+H2A pores, we fluorescently labeled H2A with Alexa Fluor 647 and tracked its entry into the cytoplasm. PMB significantly increased AF647-H2A uptake (Figures S4E and S4F), consistent with the interpretation that PMB permeabilizes the OM and IM membranes and facilitates H2A entry into bacteria. Similar trends in PI, FITC-dextran, and AF647-H2A uptake were observed in *P. aeruginosa* (Figures S5A–S5F). The cryoEM, TEM, PI, FITC-dextran, and AF647-H2A data together support the interpretation that H2A alone has little pore-forming activity toward intact cells. While PMB alone increases OM and IM permeability, the absence of pores in the vast majority of cryoEM images suggests that most of these pores are small (<5 nm) or transient, meaning that the presence of both PMB and H2A is required to form large pores.

To gain insight into how H2A augments the pores created by PMB, we tracked single-molecule localization of fluorescent H2A (AF647-H2A) via direct stochastic optical reconstruction microscopy (dSTORM) in *E. coli*. Treatment with AF647-H2A alone caused the appearance of discrete fluorophore counts in dSTORM images (Figures 3C and 3D), consistent with the binding of H2A to the OM. The addition of PMB to AF647-H2A significantly increased the number of AF657-H2A counts (Figures 3C, 3D, and S6A), with the majority forming clusters that were <50 nm and averaging 18.7 nm in diameter (Figures 3D and S6B). Clusters >110 nm in diameter were also observed at a greater frequency in cells treated with the PMB +AF647-H2A combination (Figure S6C). These data suggest that PMB increases the localization of H2A to the cell and facilitates their organization into large clusters. The broad distribution of H2A cluster sizes could reflect different stages of pore formation, with H2A cluster sizes increasing as pores widen.

### Both IM leaflets and the inner leaflet of the OM are permeabilized by H2A

A key question that our data raised was how H2A increases IM and OM permeability, but only in the presence of PMB. In the presence of PMB, H2A could gain access to the periplasm (Figure 2A) through transient or small OM pores formed by PMB. Here, H2A could have pore-forming activity toward the OM inner leaflet and IM outer leaflet that are otherwise inaccessible without PMB. We assessed the pore-forming activity of H2A toward the IM outer leaflet using *E. coli* spheroplasts, in which the OM and cell wall were removed, leaving predominantly the outer leaflet of the IM exposed,^40^ and tracking changes in PI uptake in at least 6,000 individual spheroplasts over the course of 12 min.

**Figure 4.**
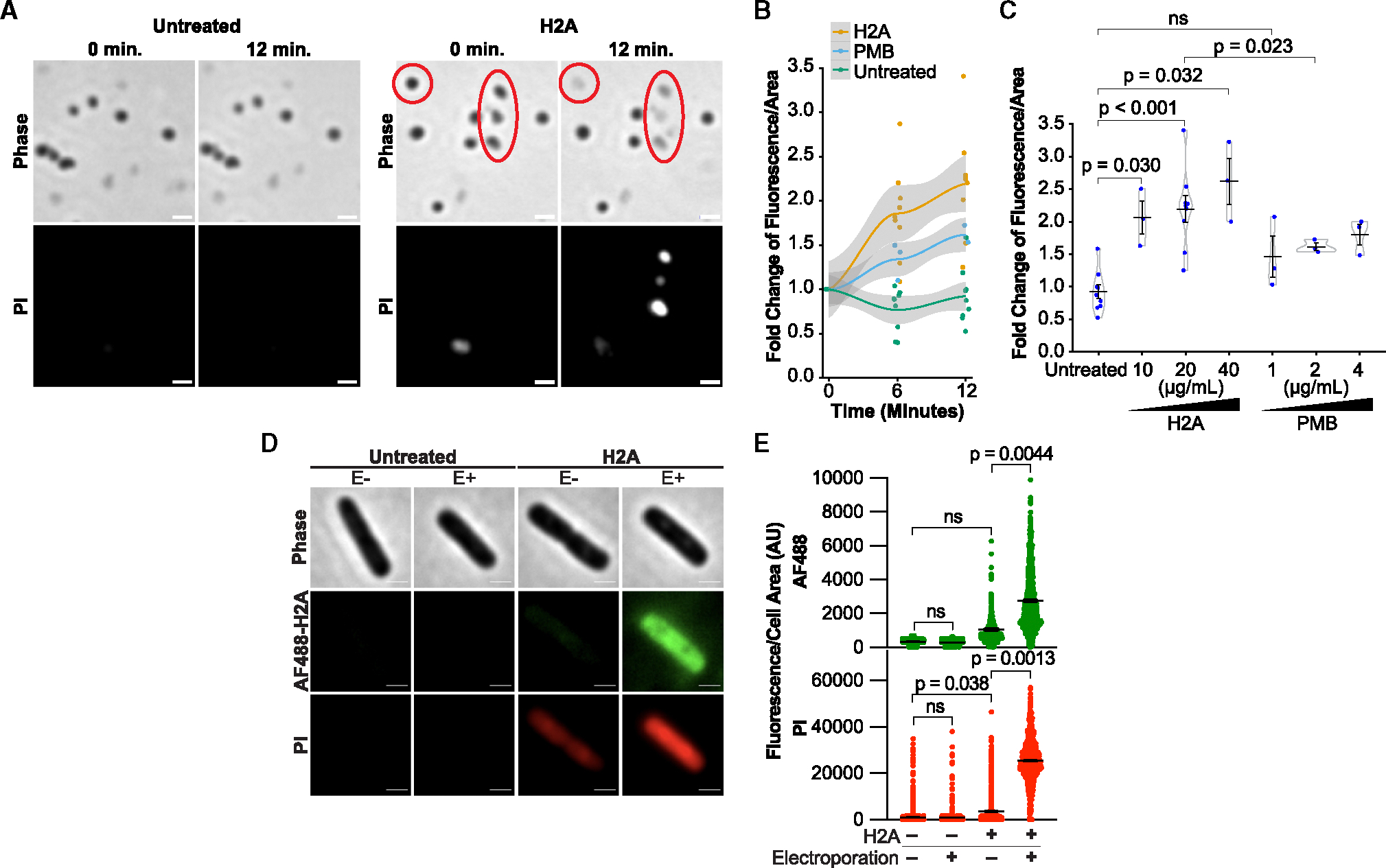
Pore-forming activity of H2A toward the IM outer leaflet and inner leaflets of the IM and OM (A) Representative phase-contrast and propidium iodide (PI) fluorescence images of *E. coli* spheroplasts pulsed with either media alone, or media containing 40 μg/mL H2A immediately after the initial time point. Red circles highlight spheroplasts that lysed during the treatment. Scale bars represent 2 μm. (B) Fold change in PI fluorescence per spheroplast area after pulsing either 20 μg/mL H2A or 2 μg/mL PMB. Shaded region indicates a local regression model using Gaussian smoothing with a confidence interval of 95%. (C) Fold change in PI fluorescence per spheroplast area at 12 min following pulsing of H2A or PMB at the indicated concentrations. Data points represent independent experiments in which at least 2,000 individual spheroplasts were analyzed. Violin plots were assembled using a Gaussian density kernel. (D and E) (D) Representative phase-contrast and fluorescence images and (E) mean intracellular fluorescence/cell area of *E. coli* 10 min following treatment with PI alone or with 25 μg/mL fluorescent H2A (AF488-H2A) and electroporated (E+) or not electroporated (E−) for each condition. Scale bars represent 1 μm. Data points represent individual cells from three independent experiments. In plots, black bars represent mean and error bars indicate SEM. *p* values in (E) were obtained using two-tailed t tests with unequal variances, and *p* > 0.05 are denoted as non-significant (ns). See also Figures S6 and S7.

We found that H2A alone caused spheroplasts to rupture and induced rapid PI uptake and membrane permeability (Figures 4A and 4B). PMB alone induced a non-significant change in PI uptake at the concentration used in our experiments (1 μg/mL) but modestly increased with higher concentrations (Figure 4C). Notably, H2A at all concentrations induced greater PI uptake than PMB. The impacts of H2A or PMB on spheroplasts were the opposite of those observed in whole cells, which contain an intact OM. Treatment of whole cells with H2A alone did not produce any significant increase in PI, FITC-dextran, or AF647-H2A fluorescence in the cytoplasm (Figures 3A, 3B, and S4A–S4F). The spheroplast data show that H2A alone permeabilizes the IM outer leaflet better than PMB and suggest that histones have differential activity toward the outer leaflets of the OM and IM.

To directly test whether H2A permeabilizes the OM and IM inner leaflets, we introduced fluorescent H2A (AF488-H2A) into the cytoplasm of *E. coli* via electroporation. Cytoplasmic AF488-H2A fluorescence confirmed H2A uptake into the electroporated cells. This uptake coincided with a significant (23-fold) increase in PI uptake (Figures 4D and 4E), indicating that H2A dramatically increased permeabilization of both the IM and OM from within the cell. This membrane permeabilization effect was not due to electroporation alone, as PI was not detected in electroporated cells without H2A (Figures 4D and 4E). The effect cannot be attributed solely to the inhibitory effects of histones on transcription, as the increase in PI uptake was observed within 10 min of H2A introduction. No significant increase in H2A uptake was detected in cells that were incubated with H2A but not electroporated (Figures 4D and 4E), but a 3-fold increase in PI uptake was observed. We attribute this modest PI uptake to the increased susceptibility of cells to H2A due to the electrocompetent preparation procedure. These data suggest that H2A permeabilizes the IM and OM inner leaflets and that H2A permeabilizes the IM outer leaflet better than PMB.

### Lipopolysaccharides inhibit the pore-forming activity of H2A in the OM

**Figure 5.**
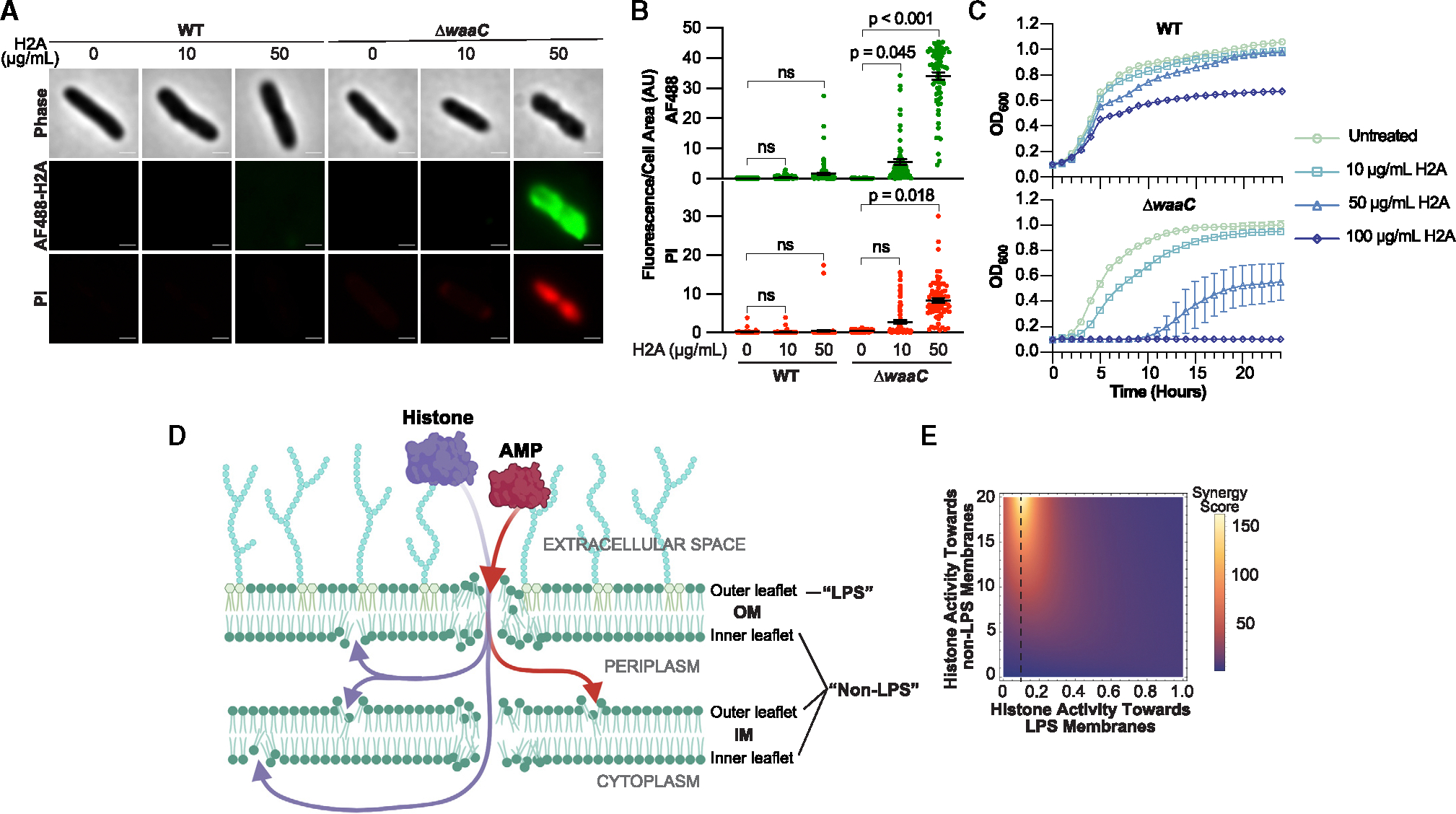
Impact of LPS on H2A pore-forming activity and orthogonal model of antimicrobial synergy (A and B) (A) Representative phase-contrast and fluorescence images and (B) mean intracellular fluorescence/cell area (AU) of wild-type (WT) and Δ*waaC E. coli* after 1-h treatments with fluorescent H2A (AF488-H2A). Scale bars represent 500 nm. Data points represent individual cells from three independent experiments. Black bars indicate mean and error bars indicate SEM. (C) Optical densities (OD_600_) of WT and *ΔwaaC E. coli* treated with the concentrations of H2A indicated. Data points represent the average of four independent experiments and error bars indicate SEM. (D) Proposed model of AMP+histone antimicrobial synergy in which AMPs enable histones to translocate past LPS-rich outer membrane (OM) into the periplasm, where histones can form pores in non-LPS membranes. (E) Antimicrobial synergy scores for a range of histone pore formation rates against LPS and non-LPS membranes. Scores were computed using a theoretical model that determines the relative concentrations of AMPs and histones in cells that are treated with the molecules individually or in combination. The histone pore formation rates are normalized by a fixed AMP pore formation rate, yielding dimensionless parameters along the *x* and *y* axes (*k*_hl_/*k*_al_ and *k*_hnl_/*k*_al_, respectively). The vertical dashed line indicates *k*_hl_/*k*_al_ = 0.1. The full derivation, parameters, and discussion of the model are in the STAR Methods and synergy model. Data points represent the average of four independent experiments and error bars indicate SEM. *p* values were obtained using two-tailed t tests assuming unequal variances and *p* > 0.05 are denoted as non-significant (ns). See also Figures S6–S8.

In Gram-negative bacteria, the bulk of the LPS is contained on the OM outer leaflet, with the IM leaflets containing significantly less LPS.^41^ From the above findings, we hypothesized that H2A has low membrane-permeabilizing activity toward the OM outer leaflet due to the presence of LPS. PMB targets LPS, which forms a protective layer on the outer leaflet of the OM.^14,42,43^ While H2A can bind to LPS,^44^ H2A may have higher activity toward other targets. We assessed the impact of H2A on membrane permeability in an *E. coli* Δ*waaC* mutant, which lacks the oligosaccharide core of LPS but retains the lipid A membrane anchor.^45^ The Δ*waaC* mutant exhibited significantly increased H2A uptake and slightly increased PI fluorescence in response to H2A at the same 10 μg/mL concentration used throughout the study (Figures 5A and 5B). These effects were intensified at a higher (50 μg/mL) concentration of H2A. In contrast, neither concentration showed increased PI fluorescence or H2A uptake in wild-type (WT) cells (Figures 5A and 5B). These data show that LPS-deficient OMs are susceptible to the membrane-permeabilizing effects of H2A.

We predicted that membrane permeabilization by H2A should hinder cell growth. Indeed, H2A treatment of the Δ*waaC* mutant delayed exponential growth and fully inhibited growth at the highest concentration tested (100 μg/mL) (Figure 5C). No growth delays were observed in the WT strain, although final cell density was lower. Thus, LPS inhibits the membrane-permeabilizing activity and growth-inhibitory effects of H2A.

### Antimicrobial synergy is due to orthogonal activity toward the membrane

Our findings support a model of AMP+histone synergy in which AMPs permeabilize the OM, which enables the uptake of AMPs and histones into the periplasmic space, where the histones can permeabilize the OM inner leaflet and IM outer leaflet (Figure 5D). We developed a theoretical model to understand how these membrane-permeabilizing activities give rise to synergy. The model simulated the entry of AMPs and histones into bacteria using a system of differential equations (schematic in Figure S6D; model derivation described in the STAR Methods and synergy model). We differentiated between pore-formation activity toward two types of membrane surfaces: the LPS-containing OM outer leaflet surface, and the OM inner leaflet and both IM leaflet surfaces, which contain far less LPS and are referred to here collectively as non-LPS surfaces. We did not consider cooperativity between AMPs and histones because no interactions between PMB and H2A were observed in solution using isothermal titration calorimetry (Figure S6E). Antimicrobial synergy scores were computed; the scores are ratios that measure the relative concentrations of AMPs and histones in the cytoplasm, where these molecules have additional disruptive activity.^7,46^ Synergy scores were assessed for a broad range of pore-forming rates toward LPS and non-LPS surfaces and plotted using dimensionless rate ratios to determine how changes in pore formation rates impact synergy (parameters in the synergy model).

We found that synergy increased when the activity of histones and AMPs were orthogonal; the maximum synergy was observed when histone activity toward LPS surfaces was low (vertical dashed line in Figure 5E) and histone activity toward non-LPS surfaces was high. Our experimental data indicate that H2A alone has low pore-forming activity toward the LPS-containing outer leaflet of the OM (Figures 3A, 3B, S4A, S4B, S4E, and S4F) and that H2A has higher pore-forming activity toward non-LPS surfaces (Figures 4A–4E and5A–5C), suggesting that the PMB+H2A combination is near a synergy peak.

Based on the prediction that antimicrobial synergy arises between histones and molecules that target the OM outer leaflet, we assessed synergy between H2A and the mammalian immunity molecule LL-37, which is found together with H2A in NETs and lipid droplets. The ability of LL-37 to form toroidal pores in the outer leaflet of the OM^6,7^ suggests that the LL-37+H2A combination should be synergistic. Indeed, treating *E. coli* with the LL-37+H2A combination (1) produced an FIC index of 0.27 (Figures S7A–S7D), (2) decreased bacterial viability by 3 orders of magnitude, (3) inhibited bacterial growth by 5 h longer than LL-37 alone, (4) produced 17.0 nm round membrane deformations (Figures S3A and S3B), and (5) caused the localization of H2A into clusters that averaged 19.5 nm in diameter (Figure S6B), with some having a diameter of ≥110 nm (Figure S6C). These data suggest that the mechanism of synergy with H2A is similar for LL-37 compared to PMB and support the hypothesis that synergy arises between molecules that differentially target non-LPS and LPS surfaces.

To further characterize our model, we evaluated the prediction that synergy disappears when H2A and AMP activity are non-orthogonal. In the Δ*waaC* mutant, the LPS truncation unmasks the membrane-permeabilizing activity of H2A toward the OM outer leaflet (Figures 5A and 5B) but does not affect PMB activity (Figure S7D), potentially due to the remaining lipid A component of LPS. The PMB+H2A combination thus has overlapping (non-orthogonal) activity toward the OM outer leaflet, which should decrease synergy toward this strain. Evidence for this is that PMB+H2A yielded an FIC index of 0.63 toward the Δ*waaC* mutant, indicating an additive rather than a synergistic antimicrobial effect (Figure S7D).

## DISCUSSION

While the antimicrobial activity of histones has long been reported, mechanistic insight has not been forthcoming. The results of the current study demonstrate that histone H2A permeabilizes all membrane surfaces except the OM outer leaflet, where H2A is inhibited by LPS. Histones alone have far more potent pore-forming activity than AMPs, as seen by their outsized activity on the spheroplasts and when electroporated into the cytoplasm. This finding directly addresses the long-standing confusion about why histones are not active at physiological ionic conditions, which can now be attributed to their activity being blocked by LPS. Given that AMPs are effective at permeabilizing the OM outer leaflet^47^ and that histones can form pores on all other membrane surfaces, the AMP+histone combination is an orthogonal and potent mechanism that can effectively disable bacteria through pore formation on all membrane surfaces. The prevalence of histones, which are widely conserved across eukaryotic organisms, and their co-occurrence with AMPs, raises the possibility that the AMP+histone combination constitutes a highly evolved antimicrobial mechanism and explains the effectiveness of this mechanism of immune defense.

The 26-nm pores created by PMB+H2A were significantly larger than what has been described for other pore-forming AMPs.^48,49^ How does H2A cause this pore formation in the presence of PMB? In the absence of PMB, H2A may be ineffective at pore formation due to its moderate binding affinity for LPS (K_D_ = 0.78 μM),^44^ leading to its sequestration and potential OM protection by LPS. The presence of PMB alone caused membrane permeabilization, as evidenced by PI and FITC-dextran uptake, but caused no detectable pores in cryo-EM images. To explain these observations, we propose that PMB causes small (<5 nm) or transient pores. These small pores allow the entry of H2A into the periplasmic space, where H2A alone can then target interior membranes containing little LPS and create large stable pores (Figure 5D). In particular, dSTORM measurements and AF647-H2A tracking support that intracellular H2A accumulation is enabled by PMB. The ability of H2A to target interior membrane surfaces is supported by the observation that spheroplasts ruptured after being treated with H2A and the increased entry of PI into H2A-treated spheroplasts relative to PMB-treated spheroplasts. A deeper mechanistic understanding of histone-mediated pore formation on interior membranes remains for future studies.

Synergistic antibiotic combinations are an important area of research and have been highly sought after due to the rise of antibiotic resistance and the ability of synergistic approaches to combat resistance.^50–54^ While numerous synergistic antimicrobial combinations have been identified, understanding how synergy arises has been limited. Our results provide a mechanistic explanation of synergy between AMPs and histones, which can be attributed directly to their orthogonal membrane targeting of LPS-containing and non-LPS membranes, respectively. We note that the LL-37 + H2A synergy index and bactericidal activity were lower than those of the PMB+H2A combination. This reduction may be attributed to differences in the LL-37 and PMB mechanisms, which could distinctly alter H2A influx and membrane sensitivity. In particular, the organization of LPS into crystalline domains potentially exposes large patches of membrane that are free of LPS, which could increase the pore-forming activity of H2A toward the outer leaflet of the OM. While the observation of synergy between H2A and either LL-37 or PMB are consistent with the orthogonal membrane targeting model, the LPS-specific effects of PMB could further enhance this synergy. In particular, our results do not rule out a cooperative interaction between histones and AMPs on membrane surfaces. Future studies using model membranes could address a potential role for molecular cooperativity.

In addition to the formation of membrane pores described here, histones can reorganize bacterial genomic DNA, thereby inhibiting transcription and replication.^6^ PMB enables H2A uptake into the cytoplasm and is thus expected to alter genome reorganization and inhibit transcription and replication. The AMP+histone combination therefore constitutes a multipronged attack on bacteria through the creation of membrane pores and disruption of transcription and replication.

The synergistic antimicrobial activity of PMB+H2A in particular could be effective against Gram-negative bacterial infections in clinical settings, supported by our *in vivo* studies. PMB and other polymyxins are already administered as antimicrobial treatments. The use of histones or a molecule that has similar effects in combination with polymyxins in clinical care settings could significantly improve antibiotic efficacy while reducing polymyxin concentration. This latter condition is advantageous because high concentrations of polymyxins cause neurotoxicity and nephrotoxicity.^55^ While our study focused on PMB and H2A, the OM-permeabilizing activity and synergy also observed with LL-37 in place of PMB suggests that differential membrane targeting can give rise to histone synergy with other AMPs. Orthogonal targeting could therefore be an effective antimicrobial strategy that would increase antimicrobial efficacy of AMPs while utilizing individual antibiotics at lower concentrations. Employing such mechanisms for treating certain bacterial infections could thus slow the progression of the antibiotic resistance crisis.

### Limitations of the study

The work here shows that large pore formation arises through synergy between full-length histone H2A and the AMPs PMB and LL-37. While the N-terminus of H2A possesses pore-forming activity,^56^ it is unclear whether the N terminus alone is sufficient to produce this synergistic effect or whether other portions of H2A are required. Similar to H2A, other histones (H1, H2B, H3, and H4) and histone-derived peptides are strongly positively charged.^7,56^ It is possible that synergy arises between these molecules and PMB or other AMPs. Consistent with this expectation, antimicrobial synergy has been observed between H3 and LL-37.^6^ Future work will need to address whether large pore formation is observed between other combinations of histones or histone-derived peptides and AMPs. Their antimicrobial synergy could lead to the establishment of a new class of antimicrobials. In addition, while antimicrobial effects have been observed toward *E. coli* and *P. aeruginosa* here, Gram-negative bacteria have diverse LPS structures and membrane properties. Future work will thus need to address whether the synergistic effects of H2A and PMB are observed across other Gram-negatives. In Gram-positive bacteria, distinct cell wall physiology may limit the synergy between H2A and PMB, as PMB efficacy is significantly reduced toward this class of bacteria. Whether and how synergy arises toward Gram-positives will thus need to be investigated. Interestingly, histone H1 alone has significant antimicrobial activity toward *Staphylococcus aureus*, although other histones do not appear to have similar activity.^57^

### RESOURCE AVAILABILITY

#### Lead contact

Correspondence and requests for materials should be addressed to Albert Siryaporn (asirya@uci.edu).

#### Materials availability

All data needed to evaluate the conclusions in the paper are present in the paper and/or the supplemental information. Raw data and strains used in this study are available upon request. This study did not generate new unique reagents.

#### Data and code availability

- Data: raw data used for statistical analyses are available in Data S1. Raw data files generated in this study are available upon request.
- Code: the custom MATLAB scripts used for processing and analyzing the fluorescence microscopy data and the custom Python scripts used for dSTORM analyses are available at Zenodo: 10.5281/zenodo.15200252.
- All other items: N/A

## STAR★METHODS

### EXPERIMENTAL MODELS

This section provides an overview and provides key details of the organisms used in this study. Further experimental details are provided below in the method details section.

#### Bacterial strains

*E. coli* experiments were performed using the wild-type *E. coli* strain MG1655 (seq).^64^ The *E. coli* MG1655 Δ*waaC* strain, which contains truncated LPS, was a gift from the Hiller lab.^14^ *P. aeruginosa* experiments otherwise were performed using the wild-type strain PAO1F.^39,58^ For the cystic fibrosis *P. aeruginosa* clinical isolate, P2m (PAmFLR02^59^), which has a mucoid phenotype, was used.

#### Galleria mellonella infection

The impact of antimicrobial agents on *P. aeruginosa* infection was assessed in a *Galleria mellonella* (wax moth larvae) model as described previously.^65^ *Galleria mellonella* larvae (Speedy Worm, Alexandria, MN) that were about ¾” in length were maintained at room temperature, fed with bedding that came with the shipment, and were used within one week of shipment arrival.

#### Murine model of corneal infection

C57BL/6J mice aged 6–8 weeks (Jackson Laboratory, Bar Harbor, ME) were used for a corneal infection model. Mouse care was performed according to UC Irvine’s Institutional Animal Care & Use guidelines. Comparable numbers of male and female mice were used for each experimental group.

#### Ethics statement

Mouse experiments were approved in protocol AUP-21–123 by the University of California Irvine’s Institutional Animal Care and Use Committee (UCI IACUC). Mice were monitored at least twice per day for signs of distress or discomfort. Any mice determined to be in distress were humanely euthanized by CO_2_ asphyxiation followed by cervical dislocation, as approved by UCI IACUC.

### METHOD DETAILS

#### Bacterial strains, growth conditions, and reagents

Bacterial strains were streaked onto LB-Miller (BD Biosciences, Franklin Lakes, NJ) petri dishes containing 2% Bacto agar (BD Biosciences) and incubated at 37°C to obtain single colonies. Single colonies were inoculated into MinA minimal medium^66^ (4.5 g KH_2_PO_4_, 10.5 g K_2_HPO_4_ 1 g (NH_4_)_2_SO_4_, and 0.5 g sodium citrate ⋅ 2H_2_O per 1 L water; 1 mM MgSO_4_) supplemented with 0.2% glucose and 0.1% casamino acids (Gibco, ThermoFisher, Waltham, MA), herein referred to as MinA+ medium. Liquid cultures were grown overnight to stationary phase at 37°C on a roller drum at 18 rpm. Overnight cultures were diluted 1:100 into fresh media and sub-cultured to an optical density at 600 nm (OD_600_) of 0.3–0.4, which is referred to here as mid-exponential phase.

The antimicrobial agents calf thymus histone H2A (Sigma-Aldrich, St. Louis, MO), human cathelicidin LL-37 (Anaspec, Fremont, CA), and polymyxin B sulfate salt (Sigma-Aldrich) were prepared fresh before experiments and used at indicated concentrations. Typical MICs for kanamycin, gentamicin, and tetracycline were estimated using the EUCAST antimicrobial wild-type distributions of microorganisms database.^33^

Histone H2A was fluorescently labeled by mixing 10 mg/mL Alexa Fluor 488 NHS Ester or Alexa Fluor 647 NHS Ester (Invitrogen, Waltham, MA) dissolved in DMSO, with 10 mg/mL H2A in 0.1 M NaHCO_3_ solution in a 1:20 ratio. LL-37 was labeled by mixing 2 mg/mL Atto 488 NHS Ester (Sigma-Aldrich) dissolved in DMSO with 2 mg/mL LL-37 in 0.1 M NaHCO_3_ solution in a 1:8.33 ratio. All solutions were stirred continuously in the dark at room temperature for 1 h, passed through PD MidiTrap G-25 columns (GE Healthcare Life Sciences, Pittsburgh, PA) to remove unreacted dye, aliquoted, and frozen at −80°C. Labeled molecules were thawed and used only once. To avoid saturation effects during imaging, labeled H2A and LL-37 were mixed with unlabeled species. The final Alexa Fluor 488-labeled H2A solution (AF488-H2A) contains 20% Alexa Fluor 488-labeled H2A and 80% unlabeled H2A. The final Alexa Fluor 647-labeled H2A solution (AF647-H2A) contains 5% Alexa Fluor 647-labeled H2A and 95% unlabeled H2A. The final Atto 488-labeled LL-37 solution (AT488-LL-37) contains 3% Atto 488-labeled LL-37 and 97% unlabeled LL-37.

#### Growth curves, checkerboard and CFU assays

Strains were cultured overnight in MinA+ medium to mid-exponential phase, diluted 1:20 into fresh medium, and treated with antimicrobial agents. For growth curves, 200 μL of bacterial cultures were immediately transferred to clear-bottom 96-well microplates (Corning, Corning, NY) that were sterile, tissue-culture treated, and composed of black polystyrene. Growth measurements were acquired every 15 min for up to 24 h using a BioTek Synergy HTX Multimode plate reader (Agilent, Santa Clara, CA) at 37°C. The reader was set to continuous orbital shaking mode at a frequency of 282 cpm (3 mm) with a 100 msec delay and 8 OD_600_ measurements taken per data point.

To calculate checkerboard fractional inhibitory concentrations, MICs were determined through OD_600_ measurements and the following equation was used: FIC = (A/MIC_A_) + (B/MIC_B_), where A and B are the MICs of each antimicrobial agent when used in combination, and MIC_A_ and MIC_B_ are MICs of each agent when used individually.^34,67^ Indexes of FIC ≤0.5, FIC ≥4, and 0.5 < FIC <4 were considered synergistic, antagonistic, and additive, respectively.^34,67^

For colony-forming unit (CFU) assays, following a 1:20 dilution into fresh medium, cultures were treated with antimicrobial agents for 1 h at 37°C on a roller drum, diluted in 10-fold serial dilutions into non-selective fresh media, plated as 10 μL droplets onto non-selective LB-Miller agar plates, incubated for 16–18 h at 37°C, and counted for single colonies.

#### *Galleria mellonella* (wax moth larvae) infection model

*P. aeruginosa* strain PAO1F was cultured to mid-exponential phase in MinA+ medium, resuspended and diluted 1:20 into PBS to 10^7^ CFU/mL, and mixed with 10 μg/mL H2A, 1 μg/mL PMB, both, or 480 μg/mL Gentamicin. 10 μL of the mixture was injected immediately into wax moth larvae at the junction of the rear left leg. Larval survival was assessed every 24 h, with live and healthy larvae showing yellow or tan coloration and visible movement, and deceased larvae exhibiting dark pigmentation, black bodily patches, and lack of movement. A high concentration of gentamicin (480 μg/mL) was necessary to achieve larvae survival for 5 days, as our use of a lower concentration (240 μg/mL) was ineffective.

#### Murine model of corneal infection

The impact of antimicrobial agents on *P. aeruginosa* infection was assessed in a murine model of corneal infection as described previously.^38,68^ Briefly, *P. aeruginosa* cells were grown to mid-exponential phase in MinA+ medium, resuspended and diluted 1:4 in PBS. Corneal epithelia of C57BL/6J mice aged 6–8 weeks (Jackson Laboratory, Bar Harbor, ME) were abraded with 3 × 5 mm scratches using a 25G needle, and 2 μL of PBS containing 5 × 10^4^ of *P. aeruginosa* strain PAO1 cells was applied topically. Antimicrobial treatments were applied 3 times per day using 10 μL droplets over the course of 48 h. The dosage of PMB was determined by identifying the minimum amount that caused a statistically significant reduction in CFUs after 48 h. H2A was supplied in a molar ratio to PMB that was comparable to *in vitro* experiments. Mice were euthanized by CO_2_ asphyxiation 48 h post-infection.

Corneal opacity was measured using a Leica MZ10 F Modular Stereo Microscope (Lecia Microsystems), gooseneck fiber optic light source, and a Leica DFC450 C camera (Lecia Microsystems) that captured images using the Leica Application Suite version 4.5 (Leica Microsystems). Opacity was quantified as performed previously^68^ using ImageJ v1.53a (NIH, Bethesda, MD). Percent opacity was defined as the average pixel intensity that was higher than that of eyes with no apparent disease, divided by the maximum pixel intensity in the image series, and normalized by cornea area. Areas of glare were removed from the analysis process. Corneal CFUs were measured by homogenizing mouse eyes in PBS using a TissueLyser II (Qiagen, Hilden, Germany) at 30 Hz for 3 min, plating in 10-fold serial dilutions onto non-selective LB-Miller agar (BD Biosciences), incubating for 16–18 h at 37°C, and counting CFUs.

#### Electron microscopy

For TEM, strains were cultured to mid-exponential phase in MinA+ medium, treated with antimicrobial agents at room temperature for 10 min and adhered to formvar-coated copper electron microscopy grids (Electron Microscopy Sciences, Hatfield, PA). Grids were washed twice in water, stained with 2% uranyl acetate for 2 min, washed again in water, and dried at room temperature overnight. Samples were imaged in a JEOL JEM-2800 transmission electron microscope containing a Schottky-type field-emission gun operating at 200 keV and a Gatan OneView CMOS camera at 4k × 4k resolution. Images were acquired using DigitalMicrograph software (Gatan Inc., Pleasanton, CA).

For cryoTEM, strains were cultured to mid-exponential phase in MinA+ medium, diluted 1:2 into fresh medium, and treated with antimicrobial agents at 37°C. *E. coli* and *P. aeruginosa* samples were treated for 15 min and 30 min, respectively, added to Lacey Carbon films on copper grids (Electron Microscopy Sciences, Hatfield, PA) that were previously glow-discharged for 70 s, and placed in a Leica EM GP2 automatic plunge freezer (Leica Microsystems, Wetzlar, Germany) that reached 95–99% humidity. Grids were blotted for 3 s prior to plunging into liquid propane. Vitrified samples were imaged on a JEOL JEM-2100F transmission electron microscope (JEOL, Peabody, MA) using a Schottky-type field-emission gun operating at 200 keV, and a Gatan OneView CMOS camera (Gatan, Pleasanton, CA) at 4k × 4k resolution. Images were acquired using SerialEM software^63^ v3.4 (University of Colorado, Boulder, CO) in low dose imaging mode. Bacterial membrane deformation and pore widths were measured along a line tangent to the membrane using ImageJ v1.53a (NIH, Bethesda, MD).

#### Fluorescence microscopy

Phase contrast and fluorescence images were acquired using a Nikon Eclipse Ti-E microscope (Nikon, Melville, NY) containing a Nikon 40X and 100X Plan Apo (1.45 N.A.) objective, a 1.5X magnifier, a Sola light engine (Lumencor, Beaverton, OR), an LED-DA/FI/TX filter set (Semrock, Rochester, NY) containing a 409/493/596 dichroic filter, a Hamamatsu Orca Flash 4.0 V2 camera (Hamamatsu, Bridgewater, NJ), and an Andor DU-897 EMCCD camera (Andor Technology, Belfast, Northern Ireland). The 474/27 nm and 525/45 nm excitation and emission filters, respectively, were used to visualize Alexa Fluor 488 and FITC-DEAE-dextran. The 575/25 nm and 641/75 nm excitation and emission filters, respectively, were used to visualize propidium iodide (PI) fluorescence. A Cy5 filter (Chroma, Bellows Falls, VT) containing 640/30 nm and 690/50 nm excitation and emission filters, respectively, and a T660lpxr dichroic was used to visualize Alex Fluor 647 fluorescence. Images were acquired using Nikon NIS-Elements version 4.5 and analyzed using AVIassembleGUI^60,61^ version 1.2c written in MATLAB R2017a (MathWorks, Natick, MA) (see ‘data and code availability‘ section for code). After treating bacteria with antimicrobial agents, 5 μL of culture was placed on 1% agarose-minimal medium pads and imaged immediately, as described previously.^69^ A minimum of 100 bacterial cells were imaged and analyzed for each experiment.

For direct stochastic optical reconstruction microscopy (dSTORM), mid-exponential cultures in MinA+ medium were diluted 1:4 into fresh medium, and treated with H2A, LL-37, or both for 40 min at 37°C on a roller drum. H2A treatments were performed at a concentration of 10 mg/mL and consisted of 95% unlabeled H2A and 5% Alex Fluor 647-labeled H2A. LL-37 treatments were performed at a concentration of 20 μg/mL and consisted of 97% unlabeled LL-37 and 3% Atto 488-labeled LL-37. Following treatment, cells were washed and resuspended in STORM buffer,^70^ which contains 143 mM β-mercaptoethanol (Sigma-Aldrich), 10% glucose, 50 mM Tris NaCl pH 8.0, 10 mM NaCl, 0.56 mg/mL glucose oxidase (Sigma-Aldrich), and 0.034 mg/mL of catalase from *Aspergillus niger* (Sigma-Aldrich), and loaded into microchamber slides containing 0.5 mm-wide channels, which were constructed by coating cover glass with 0.1% (w/v) poly-L-lysine solution (Sigma-Aldrich) that was diluted 1:100 in ethanol for 20 min, drying at 100°C for 10 min, and attaching to slides using double-sided tape. Fresh STORM buffer was flowed in after 5 min to wash away cells that were not adhered to the cover glass surface.

dSTORM imaging was performed using a custom total internal refection (TIRF) Nikon TE200 microscope containing a Nikon 100X Apo TIRF (1.49 N.A.) objective, Ti:Sapphire 488 nm or 640 nm laser (Coherent, Santa Clara, CA), QuantEM 512SC EMCCD camera (Photometrics, Tucson, Arizona), a 432/515/595/681/809 nm penta-band bandpass filter, and 405/488/561/635 nm dichroic beamsplitter (Semrock). For each cell, 2,000 frames were acquired and reconstructed into a single image using RapidSTORM software version 3.0^62^ using an input pixel size of 150 nm, 500 nm PSF FWHM, and an output resolution of 10 nm/pixel, consistent with previous analyses.^71,72^ For images in Figure 3C, the output pixels sizes were set to represent 1 nm/pixel. Quantification of fluorescent clusters was performed using scripts that were written in Python. We utilized the libraries skimage (v0.19.3), scipy (v1.10.0), pandas (v1.5.3), numpy (v1.23.5), matplotlib (v3.7.0) and PIT (v9.4.0) for image processing and plotting (see ‘data and code availability‘ section for code).

#### Membrane permeability assays

To visualize bacterial membrane permeability, mid-exponential cells cultured in MinA+ medium were diluted 1:20 into fresh medium, treated with antimicrobial agents for 1 h at 37°C, supplemented with 30 μM PI for 15 min, immobilized on 1% agarose pads containing MinA+ medium, and imaged immediately using fluorescence microscopy. To estimate bacterial pore sizes, mid-exponential strains cultured in MinA+ medium were diluted 1:20 into fresh medium, treated with antimicrobial agents for 45 min at 37°C on a roller drum, mixed with 0.25 mg/mL of 40 kDa or 150 kDa FITC-DEAE-dextran (Sigma-Aldrich) for 15 min, washed twice in MinA+ medium, immobilized onto 1% agarose pads containing MinA+ medium, and immediately visualized using fluorescence microscopy. Dextran diameters were based on Stokes’ radii reported in the manufacturer’s technical documents (Fluorescein Isothiocyanate-Dextran, https://www.sigmaaldrich.com/US/en/technical-documents/technical-article/cell-culture-and-cell-culture-analysis/cell-based-assays/fluorescein-isothiocyanate-dextran, Millipore Sigma), with 40 kDa and 150 kDa dextran corresponding to 9 and 17 nm pores, respectively. To assess the uptake of AF647-H2A, mid-exponential strains cultured in MinA+ medium were diluted 1:5 into fresh medium, treated for 45 min or 1 h for *E. coli* and *P. aeruginosa*, respectively, with AF647-H2A alone or in combination with PMB, and immobilized onto 1% agarose pads.

#### Bacterial spheroplast time-lapses

Bacterial spheroplasts were prepared based on previous protocols.^40,73^ Briefly, *E. coli* were cultured for 18 h in LB-Miller Broth (BD Biosciences), washed twice and resuspended in Cation-Adjusted Mueller Hinton Broth (CA-MHB) (BD Biosciences), incubated for 2 h at 37°C with shaking at 180 rpm, washed using 0.03 M Tris-HCl pH 8.0 (Tris buffer), resuspended in Tris buffer containing 20% sucrose, 0.012 mg/mL ethylenediaminetetraacetic acid (EDTA), and 0.04 mg/mL lysozyme (Sigma-Aldrich), incubated at 30°C for 1 h with shaking at 180 rpm, and resuspended in Tris buffer containing 20% sucrose. Immediately before imaging, spheroplasts were diluted 1:10 into Tris buffer containing 20% sucrose, 1 mM MgSO_4_, and 10 μM PI, injected into 0.5 × 2.4 cm microchannels that were assembled using cover glass and double-sided tape, centrifuged at 70 g for 3 min, and imaged immediately. Phase contrast and fluorescence images were acquired at room temperature every 3 min for 21 min. Following the initial 5 min, Tris buffer containing 20% sucrose, 1 mM MgSO_4_, 10 μM PI, and antimicrobial agents were pulsed into the channel. Images were analyzed using AVIassembleGUI^60,61^ version 1.3 written in MATLAB R2023b (see ‘data and code availability‘ section for code).

#### Histone electroporation

Electrocompetent *E. coli* were prepared by culturing in SOB medium^74^ to mid-exponential phase, washing with 10% glycerol four times, resuspending to an OD_600_ of 0.2, and freezing at −80°C. *E. coli* were thawed, supplemented with 1 μM MgSO_4_, electroporated with 10 μg/mL of H2A using a Bio-Rad Micropulser (Bio-Rad, Hercules, CA), resuspended in cold MinA+ medium, incubated for 10 min at room temperature, supplemented with 30 μM PI, immobilized on 1% agarose pads containing MinA+ medium, and imaged immediately using fluorescence microscopy.

#### Theoretical model of AMP-Histone synergy

We constructed a predictive model that describes the dynamics of AMPs and histones in a Gram-negative bacterium, which is derived in full detail in the synergy model and simulated using Mathematica. Briefly, AMPs and histones are initially present in the extracellular space and translocate to the periplasm and cytoplasm through membrane pores. We distinguish between two types of membrane surfaces: LPS membranes, in particular the outer leaflet of the OM; and the inner leaflet of the OM and both leaflets of the IM, referred to as non-LPS membranes. The synergy score is computed based on the concentrations of AMPs and histones in the cytoplasm following a fixed simulation time and compares the concentrations of AMPs and histones when cells are treated with the molecules individually or in combination:

$$S=\frac{{\left[A_{\text{c}}\right]}_{\text{combined}}+{\left[H_{\text{c}}\right]}_{\text{combined}}}{{\left[A_{\text{c}}\right]}_{\text{individual}}+{\left[H_{\text{c}}\right]}_{\text{individual}}},$$

where $\left[A_{\text{c}}\right]$ and $\left[H_{\text{c}}\right]$ refer to AMPs or histones in the cytoplasm, respectively, and individual and combined refer to the AMPs or histones that are supplied individually or in combination in the initial conditions, respectively. The concentration is assessed in the cytoplasm because their presence here decreases cell viability (Figures 1A and 1B; Figures S1A and S4A, S4B, and S4E, and S4F;^6^). If the concentrations inside the cytoplasm are greater in combination than individually, a synergy score of greater than one results. Higher scores represent greater levels of synergy. Synergy was assessed for a broad range of histone and AMP pore formation rates toward LPS and non-LPS membranes.

#### Isothermal titration calorimetry

ITC was performed by the Sanford Burnham Prebys Protein Production and Analysis Facility (La Jolla, CA) using a Low Volume Affinity ITC calorimeter (TA Instruments, New Castle, DE). Aliquots containing 600 mM PMB were injected into a cell containing 100 mM H2A. The experiments were performed at 25°C in PBS and 20 injections in total were performed. ITC data were processed using NanoAnalyze software (TA Instruments).

### QUANTIFICATION AND STATISTICAL ANALYSIS

Relevant statistical details are provided alongside figures in figure legends. Datasets containing experimental groups with large variations (greater than two standard deviations from the mean) between biological replicates were typically repeated to assess reproducibility. Large variations were included if they were replicated in repeated experiments. Data points were considered outliers and excluded if the variation was not reproduced. Statistical analysis was performed using unpaired one- or two-tailed t-tests with unequal variances using GraphPad Prism version 9.5 or R version 4.3.1. Statistical significance was defined as a *p* value of <0.05. Raw data used for statistical analyses are available in the Source Data file (Data S1).

## Supplementary Material

1

2

3

Supplemental information can be found online at https://doi.org/10.1016/j.celrep.2025.115658.

## Figures and Tables

**KEY RESOURCES TABLE:** 

REAGENT or RESOURCE	SOURCE	IDENTIFIER

Strains		

E. coli MG1655 (seq)	Goulian lab (University of Pennsylvania, Philadelphia, PA)	CGSC #7740
E. coli MG1655 Δ*waaC*	S. Hiller (University of Basel, Basel, Switzerland)	Manioglu et al.^14^
*P. aeruginosa* strain PAO1F	Pearlman lab (University of California Irvine, Irvine, CA)	Vareechon et al.^39^ and Vance et al.^58^
*P. aeruginosa* P2m PAmFLR02	K. Whiteson lab (University of California Irvine, Irvine, CA)	Quinn et al.^59^

Chemicals, peptides, and recombinant proteins		

Alexa Fluor 488 NHS Ester	Fisher Scientific (Waltham, MA)	A20000
Alexa Fluor 647 NHS Ester	Fisher Scientific (Waltham, MA)	A20006
Atto 488 NHS Ester	Sigma-Aldrich (St. Louis, MO)	41698-1MG-F
FITC-DEAE-Dextran (40kDa)	Sigma-Aldrich (St. Louis, MO)	01649-1G
FITC-DEAE-Dextran (150kDa)	Sigma-Aldrich (St. Louis, MO)	75005-1G
Gentamicin sulfate	Sigma-Aldrich (St. Louis, MO)	G1914-250MG
Histone H2A from calf thymus	Sigma-Aldrich (St. Louis, MO)	H9250-100MG
Human Antimicrobial Peptide LL-37	Anaspec, Fremont, CA	AS-61302
Kanamycin	Sigma-Aldrich (St. Louis, MO)	60615-5G
Polymyxin B sulfate salt	Sigma-Aldrich (St. Louis, MO)	L6876-5G
Tetracycline hydrochloride	Sigma-Aldrich (St. Louis, MO)	T7660-25G

Experimental models: Organisms/strains		

C57BL/6J mice aged 6-8 weeks	Jackson Laboratory, Bar Harbor, ME	N/A
*Galleria mellonella* (Wax Moth Larvae)	Speedy Worm, Alexandria, MN	N/A

Software and algorithms		

AVIassembleGUI version 1.2c	Zenodo: https://doi.org/10.5281/zenodo.15200252	Siryaporn et al.^60^ and Perinbam et al.^61^
DigitalMicrograph software	https://www.gatan.com/resources/software Gatan Inc., Pleasanton, CA	N/A
EUCAST antimicrobial wild-type distributions of microorganisms database	https://mic.eucast.org/search/	Leclercq et al.^33^
GraphPad Prism version 9.5	https://www.graphpad.com/ GraphPad Software, Boston, MA	N/A
ImageJ v1.53a	https://imagej.net/ij/ NIH, Bethesda, MD	N/A
Leica Application Suite version 4.5	https://www.leica-microsystems.com/products/microscope-software/p/leica-application-suite/downloads/ Leica Microsystems, Wetzlar, Germany	N/A
Mathematica	https://www.wolfram.com/?source=nav Wolfram, Champaign, IL	N/A
NanoAnalyze	https://www.tainstruments.com/itcrun-dscrun-nanoanalyze-software/ TA Instruments, New Castle, DE	N/A
Nikon NIS-Elements version 4.5	https://www.microscope.healthcare.nikon.com/products/software/nis-elements Nikon Instruments, Melville, NY	N/A
R version 4.3.1	https://www.r-project.org/ R Foundation, Vienna, Austria	N/A
RapidSTORM software version 3.0	http://stevewolter.github.io/rapidSTORM/	Wolter et al.^62^
SerialEM software v3.4	https://bio3d.colorado.edu/SerialEM/download.html University of Colorado, Boulder, CO	Mastronarde^63^

## Highlights

- Histone H2A and polymyxin B (PMB) together form lethal 26 nm pores in bacterial membranes
- Pore-forming activity of H2A is inhibited by lipopolysaccharide; PMB removes this inhibition
- Similar synergistic and bactericidal effects observed between H2A and LL-37
- Histones + antimicrobial peptides could constitute a novel synergistic antibiotic strategy
