# Supplementary material for: Bactericidal activity of mammalian histones is caused by large membrane pore formation
Source: Cell Rep. Author manuscript; Available in PMC 2025 Jun 16. (PMC12168145; doi:10.1016/j.celrep.2025.115658)
Supplement: 1 [file NIHMS2085648-supplement-1.pdf]

Cell Reports, Volume *44*

Supplemental information

Bactericidal activity of mammalian histones is caused by large membrane pore formation

Leora Duong, Yonghan Wu, Summer J. Kasallis, Serena Abbondante, Paul J. Hurst, Michaela E. Marshall, Katherine McCarthy, Babu J.N. Reddy, Jean-Louis Bru, Kumar Perinbam, Eric Pearlman, Joseph P. Patterson, Steven P. Gross, and Albert Siryaporn

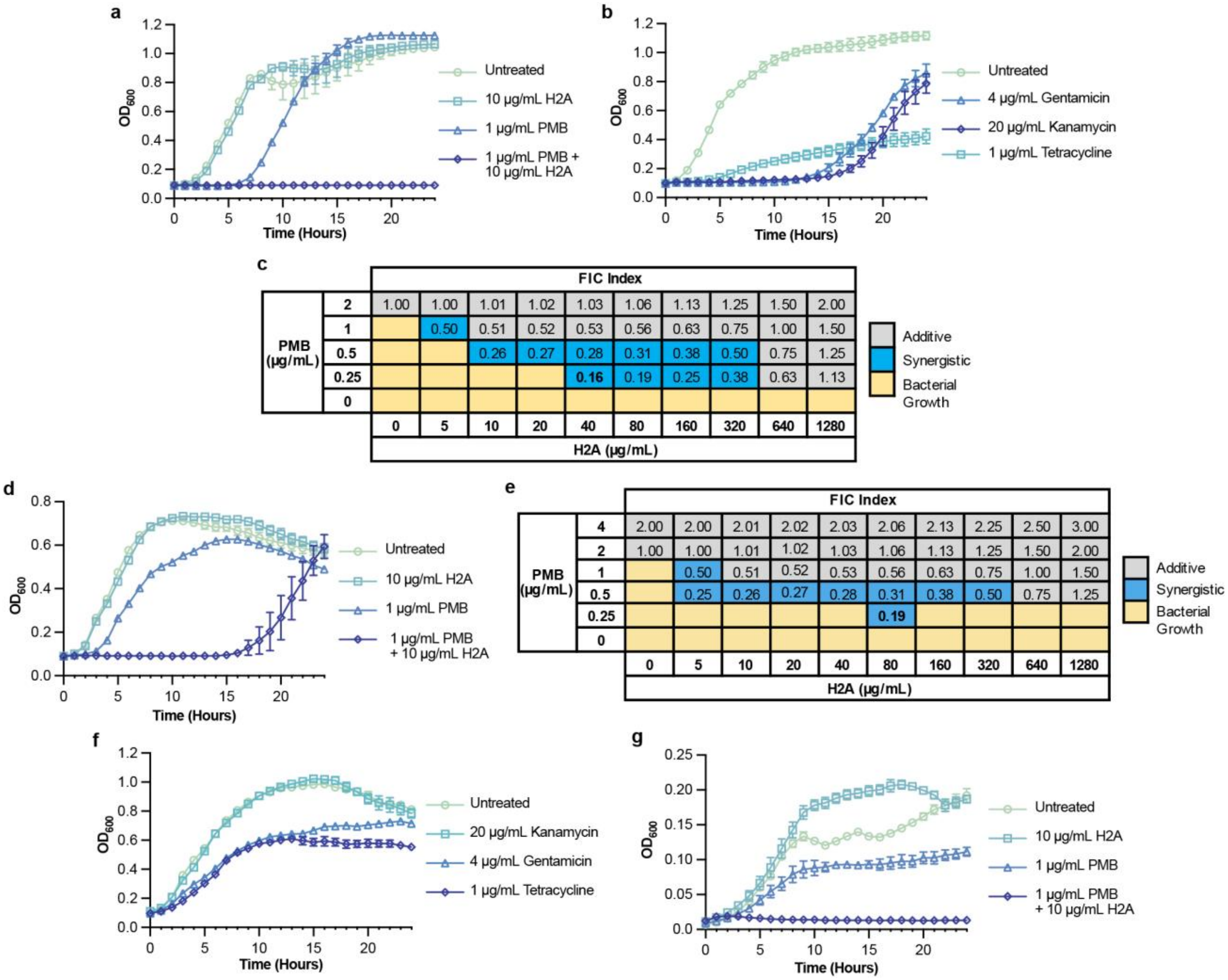

Figure S1. Impact of PMB+H2A on growth *in vitro* against *P. aeruginosa* and *E. coli* and measurements of synergy (related to Fig. 1)

(a) Optical densities (OD_{600}) of *E. coli* untreated or treated with PMB, H2A, or both at the indicated concentrations. (b) Optical densities of *E. coli* untreated or treated with kanamycin, tetracycline, or gentamicin at the indicated concentrations. (c) Checkerboard assay to assess antimicrobial synergy between PMB and H2A towards *E. coli*. FIC values are displayed for conditions in which no growth was observed after 24 hours. Values are categorized as synergistic (less than or equal to 0.5) or additive (values above 0.5). (d) Optical densities of *P. aeruginosa* either untreated or treated with PMB, H2A, or both at the indicated concentrations. (e) Checkerboard assay to assess antimicrobial synergy between PMB and H2A towards *P. aeruginosa* after 8 hours. (f) Optical densities of *P. aeruginosa* either untreated or treated with kanamycin, tetracycline, or gentamicin at the indicated concentrations. (g) Optical densities of the cystic fibrosis clinical isolate *P. aeruginosa* P2m untreated or treated with H2A, PMB, or both, at the indicated concentrations. Data points in optical density plots indicate the average of 4 independent experiments and error bars indicate standard error of the mean (SEM).

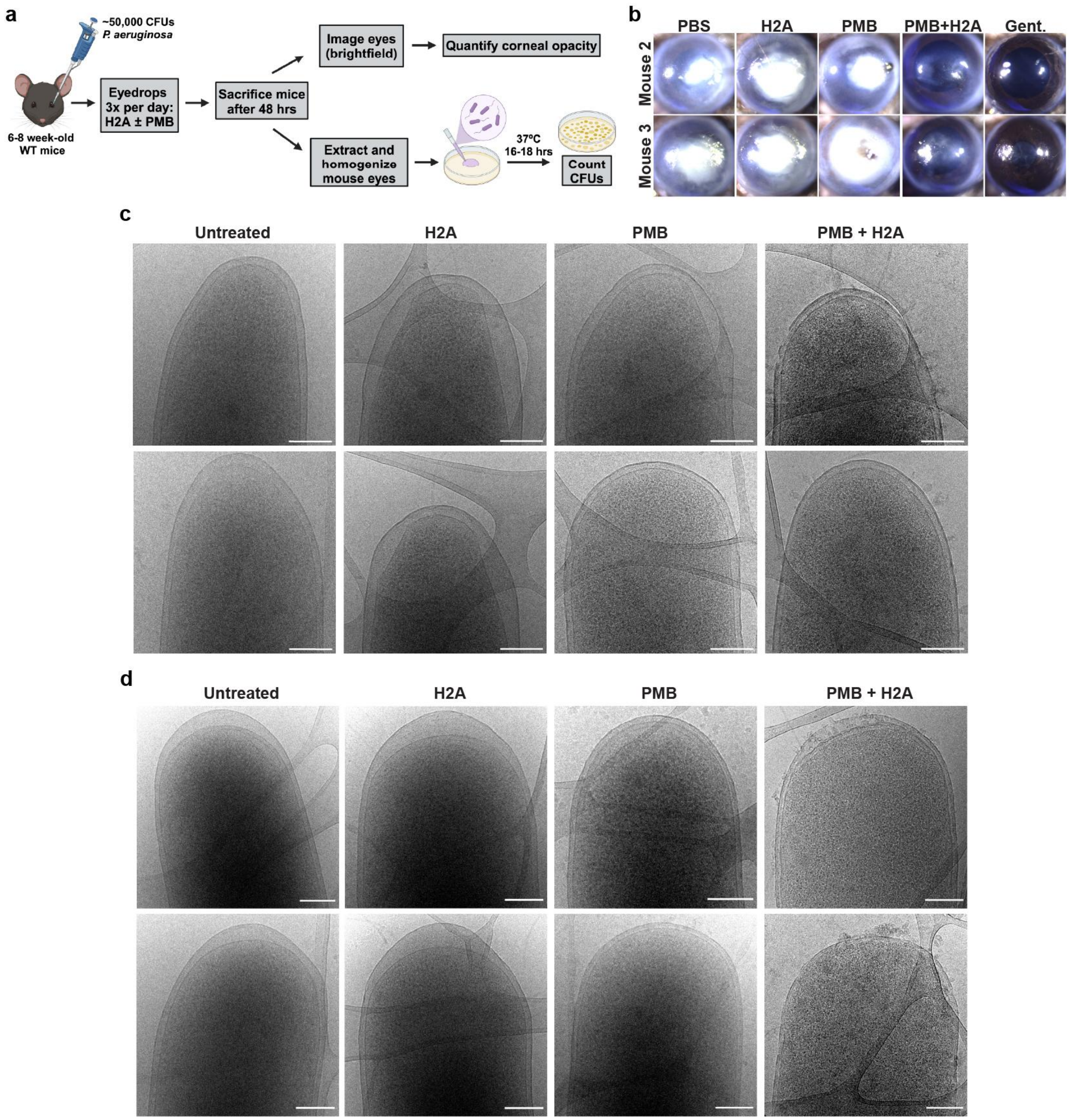

Figure S2. Impact of PMB+H2A on *P. aeruginosa* infection *in vivo* and on pore formation in bacterial membranes using cryoEM (related to Figs. 1 and 2)

(a) Schematic of murine corneal infection experiments. (b) Representative brightfield images of mouse corneas 48 hours after infection with *P. aeruginosa* and treatment with either PBS, 10 µg H2A, 500ng PMB, both PMB and H2A, or 30 µg gentamicin. (c) Representative cryoEM images of *P. aeruginosa* cells that were untreated or treated for 30 minutes with 10 µg/mL H2A, 1 µg/mL PMB, or both. (d) Representative cryoEM images of *E. coli* that were either untreated or treated with 10 µg/mL H2A, 1 µg/mL PMB, or both, for 15 minutes. Scale bars represent 200 nm. Data from panels b,c,d are additional data from experiments performed for Fig. 1d,1h, and 2a in the main text, respectively.

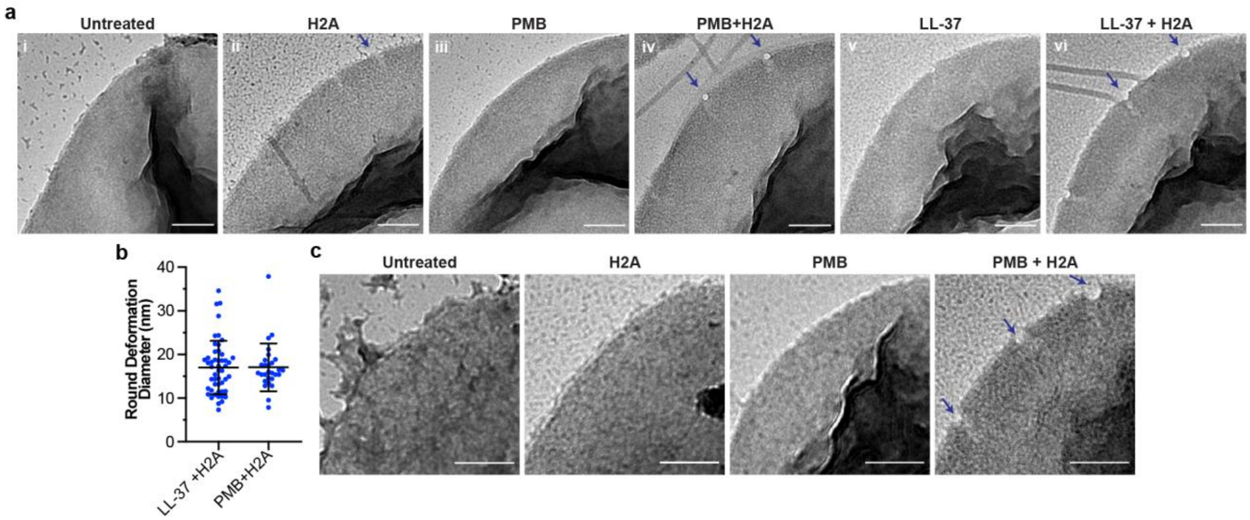

Figure S3. Membrane deformations observed in *E. coli* and *P. aeruginosa* using transmission electron microscopy (related to Fig. 2)

(a) Representative transmission electron microscopy (TEM) images of *E. coli* (i) untreated, or treated with (ii) 10 µg/mL H2A, (iii) 1 µg/mL PMB, (iv) PMB and H2A, (v) 20 µg/mL LL-37, or (vi) LL-37 and H2A. (b) Mean diameters of round deformations observed in TEM images from the complete set of experiments performed for panel a. Data points represent individual deformations. (c) Representative transmission electron microscopy images of *P. aeruginosa* either untreated or treated with 10 µg/mL H2A, 1 µg/mL PMB, or both. Arrows highlight high contrast round membrane deformations. Scale bars represent 100 nm.

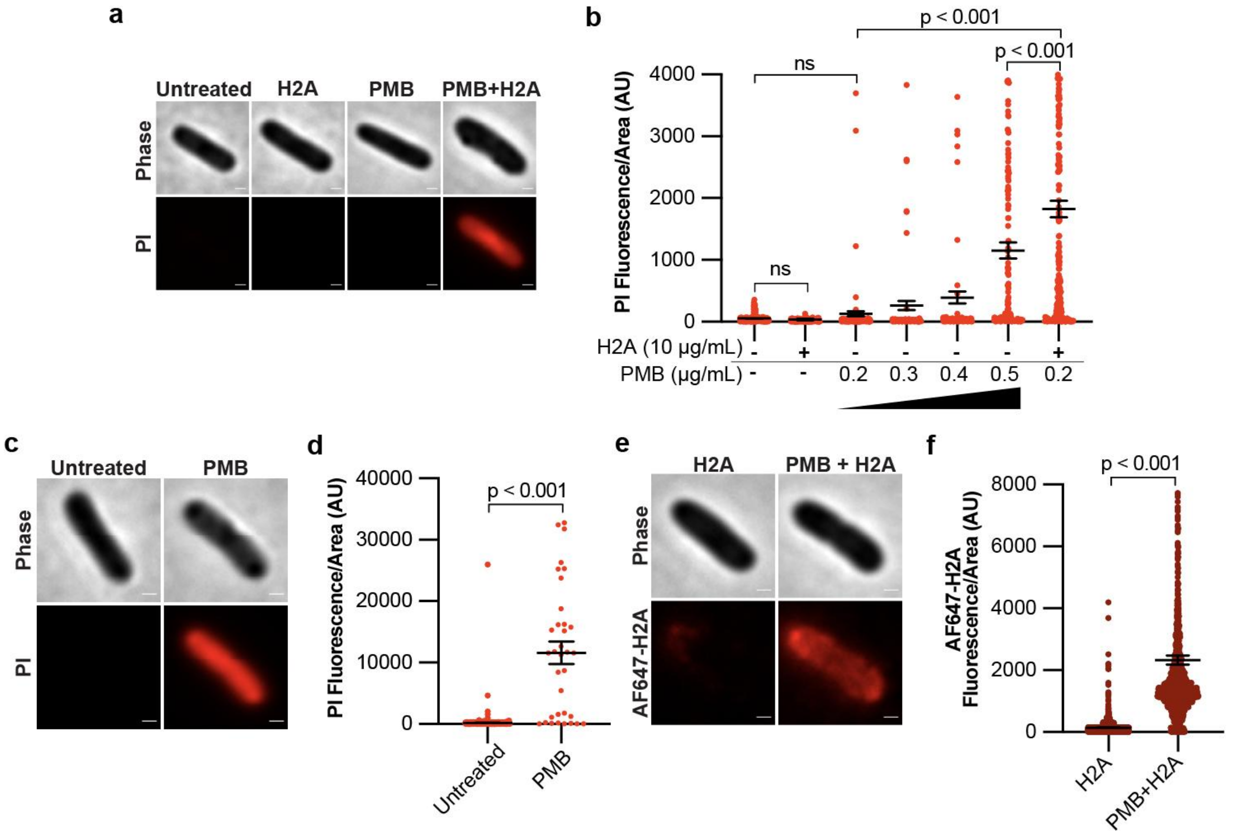

Figure S4. Impact of PMB+H2A on *E. coli* membrane permeability changes and on H2A uptake into the *E. coli* cytoplasm (related to Fig. 3)

(a) Representative phase-contrast and propidium iodide (PI) fluorescence images of *E. coli* following a 1-hour treatment of 10 µg/mL H2A, 0.2 µg/mL PMB, or both. Scale bars represent 500 nm. (b) Mean intracellular PI fluorescence/cell area (AU) of *E. coli* following a 1-hour treatment of H2A, PMB, or both, at indicated the concentrations. A small subset of data points are above the vertical axis maximum and are not displayed; full data are in Source Data. (c) Representative phase-contrast and PI fluorescence images and (d) mean intracellular PI fluorescence/cell area (AU) of *E. coli* following a 1-hour treatment with 1 µg/mL PMB. (e) Representative phase-contrast and AF647-H2A fluorescence images and (f) mean intracellular AF647-H2A fluorescence/cell area (AU) of *E. coli* following a 1-hour treatment with 10 µg/mL AF647-H2A alone or with 1 µg/mL PMB. Scale bars represent 500 nm. Data points represent individual bacterial cells. Black bars represent mean and error bars indicate SEM. Two tailed t-tests with unequal variances were performed, with *p* values >0.05 denoted as nonsignificant (ns). Note that different fluorescence acquisition times used between the panel groups a-b and c-d.

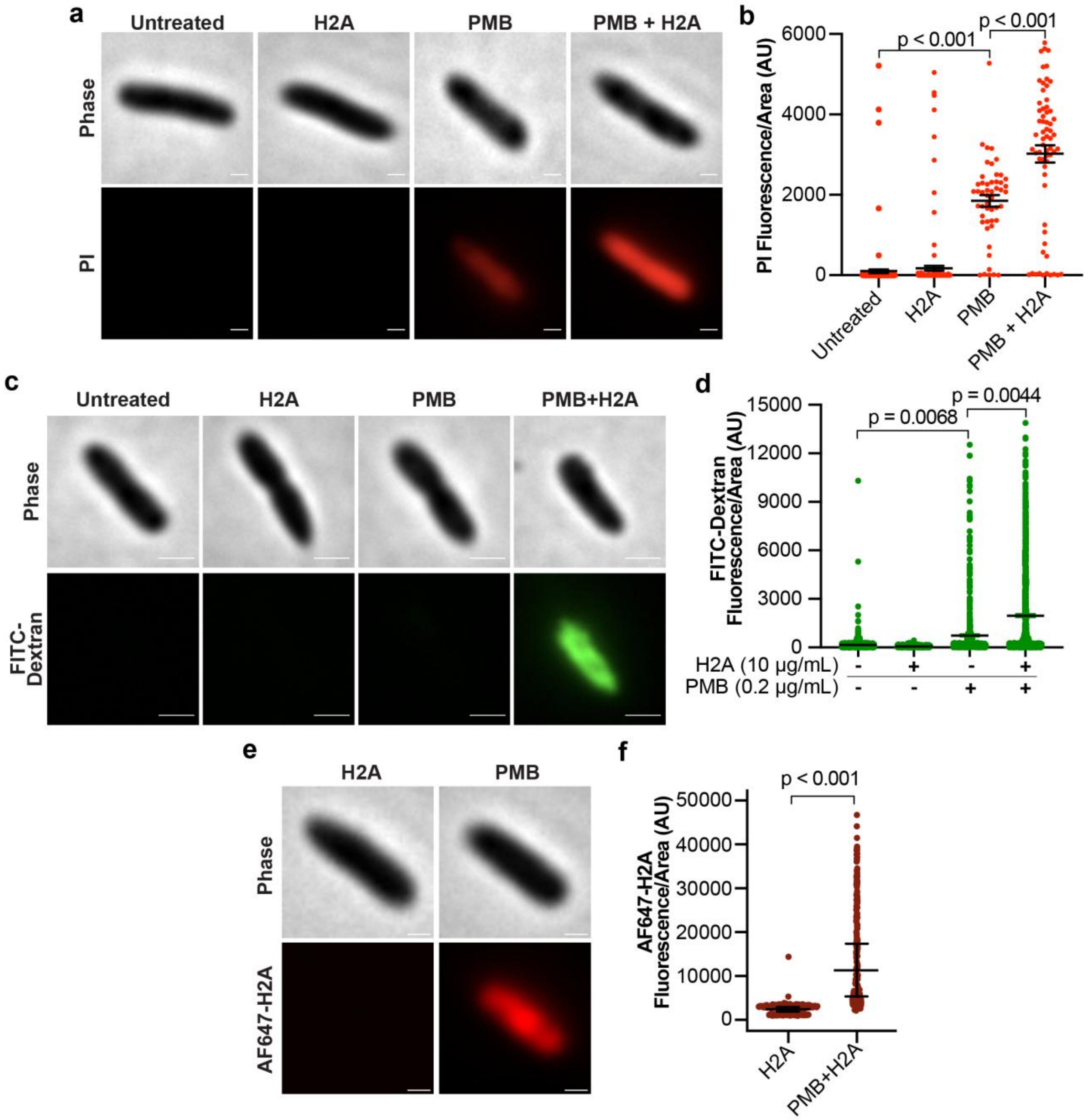

Figure S5. Impact of PMB+H2A on *P. aeruginosa* membrane permeability (related to Fig. 3)
(a) Representative phase-contrast and PI fluorescence images and (b) associated mean intracellular PI fluorescence / cell area (AU) of *P. aeruginosa* following a 1-hour treatment of 10 µg/mL H2A, 1 µg/mL PMB, both, or untreated. Scale bars represent 500 nm. (c) Representative phase-contrast and FITC-dextran fluorescence images and (d) associated mean intracellular fluorescence /cell area (AU) of *P. aeruginosa* following treatments of 10 µg/mL H2A, 1 µg/mL PMB, both, or untreated and labeled with 17 nm diameter FITC-dextran. Scale bars represent 1 µm. Data points in panels b and d represent individual bacterial cells from duplicate and triplicate experiments, respectively. Black bars represent mean and error bars indicate SEM. *p* values were determined using two-tailed t-tests assuming unequal variances. (e) Representative phase-contrast and AF647-H2A fluorescence images and (f) mean intracellular AF647-H2A fluorescence/cell area (AU) of *P. aeruginosa* following a 1-hour treatment with 10 µg/mL AF647-H2A alone or with 1 µg/mL PMB. Scale bars represent 500 nm. Data points represent individual bacterial cells. Black bars represent the mean of triplicate experiments and error bars indicate SEM. Two tailed t-tests with unequal variances were performed.

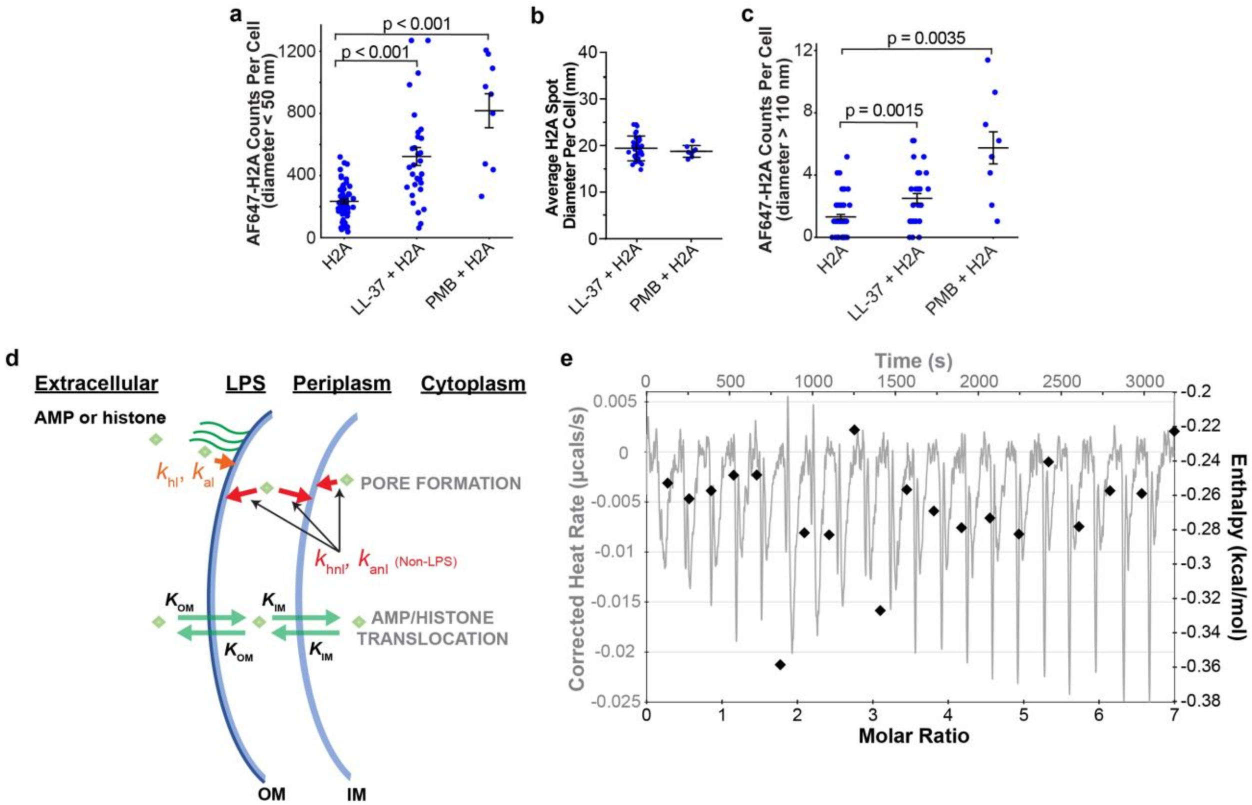

Figure S6. Clustering of H2A molecules in *E. coli* and schematic of predictive AMP+histone synergy model (related to Figs. 3-5)

(a) Number of AF647-H2A fluorescent spots per cell that were less than 50 nm in *E. coli* treated with either 10 µg/mL AF647-H2A alone or in combination with 20 µg/mL LL-37 or 1 µg/mL PMB. (b) Mean spot diameters per cell of AF647-H2A in dSTORM images for spots that were less than 50 nm. (c) Number of AF647-H2A fluorescent spots per cell that were greater than 110 nm for the same set of data in panels a and b. Data points represent individual cells for experiments that were performed in at least biological triplicate. Black bars represent mean and error bars indicate SEM. *p* values were determined using two-tailed t-tests assuming unequal variances. (d) Schematic indicating bacterial compartments, membranes, and associated parameters of the predictive AMP+histone synergy model. (e) Heat rate (grey lines) and enthalpies (black diamonds) measured using isothermal titration calorimetry of PMB+H2A for a range of molar ratios. The color of the axes correspond to the color of the data on the plot.

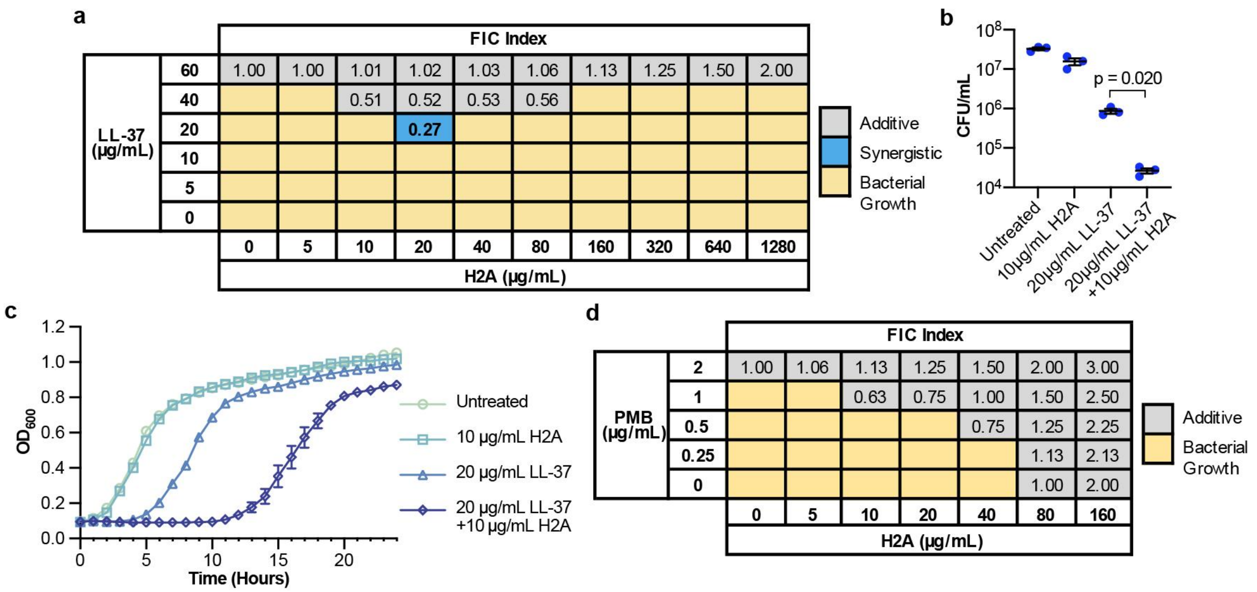

Figure S7. Impact of LL-37+H2A on growth *in vitro* and assessments of synergy (related to Figs. 3-5)

(a) Checkerboard assays, (b) CFUs, and (c) optical densities of *E. coli* treated with H2A, LL-37, both, or untreated (n=4 for each condition). (d) Checkerboard assay to assess antimicrobial synergy between PMB and H2A towards the *E. coli* Δ*waaC* mutant after 24 hours. *p* values were determined using two-tailed t-tests assuming unequal variances.

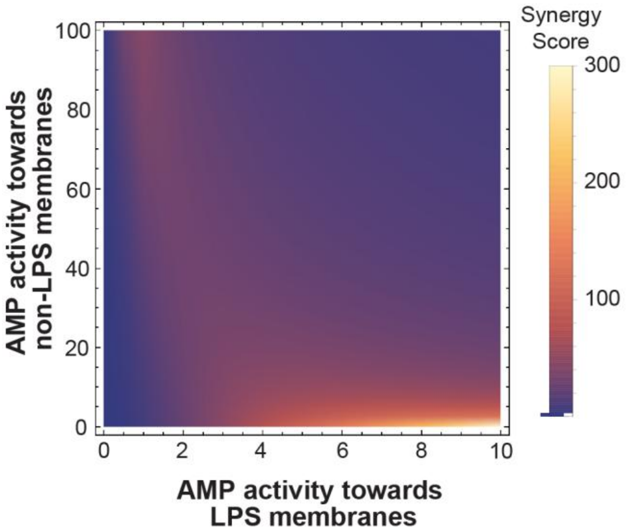

Figure S8. Synergies determined by the predictive AMP+histone model (related to Fig. 5)
Antimicrobial synergy scores for a range of AMP pore formation rates towards LPS and non-LPS membranes. The AMP pore formation rates are normalized by a fixed histone pore formation rate of k_{hl}= 0.002 s^{-1}, yielding a dimensionless value along the x and y axes (k_{al}/k_{hl} and k_{anl}/k_{hl}, respectively). The full derivation, parameters, and discussion of the model are in the Methods and Synergy Model section in the Supplementary Information.
