# Supplementary material for: Bactericidal activity of mammalian histones is caused by large membrane pore formation
Source: Cell Rep. Author manuscript; Available in PMC 2025 Jun 16. (PMC12168145; doi:10.1016/j.celrep.2025.115658)
Supplement: 3 [file NIHMS2085648-supplement-3.pdf]

Supplemental Methods S1

Synergy Model

Here we describe: 1) the kinetics of AMP and histone translocation across different membranes and cellular compartments, 2) how membrane pore formation is modeled, 3) the computation of synergy, 4) the potential involvement of cooperativity, and 4) simulation conditions.

AMP and histone translocation kinetics

AMP kinetics. The translocation of AMPs between the extracellular space, periplasm, and cytoplasm (**Fig. S6d in Supplemental Information**) can be described generally using first-order kinetics. The kinetics of translocation between the extracellular space and the periplasm is determined by K_{OM}, which represents the rate of AMP translocation across the outer membrane (OM). Similarly, translocation between the periplasm and cytoplasm is determined by K_{IM}, which represents the rate of AMP translocation across the inner membrane (IM). The AMP concentration in the extracellular space is determined by balance of AMP translocation through OM pores from the extracellular space to the periplasm and in the reverse direction, which is described by:

$$\frac{\mathrm{d}[A_{\mathrm{ex}}]}{\mathrm{d}t} = K_{\mathrm{OM}}([A_{\mathrm{p}}] - [A_{\mathrm{ex}}]), \tag{1}$$

where $[A_{\mathrm{ex}}]$ and $[A_{\mathrm{p}}]$ represent the concentration of AMPs in the extracellular and periplasmic spaces, respectively. The process of pore formation is discussed in the *Pore formation* section below.

The AMP concentration in the periplasm is determined by the influx of AMPs from the extracellular space and cytoplasm, which is offset by their efflux from the periplasm through pores in the OM and IM:

$$\frac{\mathrm{d}[A_{\mathrm{p}}]}{\mathrm{d}t} = K_{\mathrm{OM}}[A_{\mathrm{ex}}] - (K_{\mathrm{IM}} + K_{\mathrm{OM}})[A_{\mathrm{p}}] + K_{\mathrm{IM}}[A_{\mathrm{c}}], \tag{2}$$

where $[A_{\mathrm{c}}]$ is the concentration of AMPs in the cytoplasm.

The AMP concentration in the cytoplasm is determined by the influx of AMPs from the periplasm and their efflux from the cytoplasm through pores in the IM, which is described by:

$$\frac{\mathrm{d}[A_{\mathrm{c}}]}{\mathrm{d}t} = K_{\mathrm{IM}}([A_{\mathrm{p}}] - [A_{\mathrm{c}}]). \tag{3}$$

Histone kinetics. The translocation of histones across the extracellular, periplasmic, and cytoplasmic spaces can be described using analogous equations in which terms representing the concentration of AMPs have been replaced by those representing histones:

$$\frac{\mathrm{d}[H_{\mathrm{ex}}]}{\mathrm{d}t} = K_{\mathrm{OM}}([H_{\mathrm{p}}] - [H_{\mathrm{ex}}]) \tag{4}$$

$$\frac{\mathrm{d}[H_{\mathrm{p}}]}{\mathrm{d}t} = K_{\mathrm{OM}}[H_{\mathrm{ex}}] - (K_{\mathrm{IM}} + K_{\mathrm{OM}})[H_{\mathrm{p}}] + K_{\mathrm{IM}}[H_{\mathrm{c}}] \tag{5}$$

$$\frac{\mathrm{d}[H_{\mathrm{c}}]}{\mathrm{d}t} = K_{\mathrm{IM}}([H_{\mathrm{p}}] - [H_{\mathrm{c}}]), \tag{6}$$

where $[H_{\mathrm{ex}}]$, $[H_{\mathrm{p}}]$, and $[H_{\mathrm{c}}]$ represent the concentration of histones in the extracellular space, periplasm, and cytoplasm, respectively.

Pore formation

The process of pore formation in the OM and IM are determined by the concentration of AMPs and histones in the vicinity of these surfaces. We distinguish between pore formation on two types of membrane surfaces: LPS surfaces of the OM outer leaflet, which contains abundant LPS; and non-LPS surfaces on the OM inner leaflet and both leaflets of the IM, which contain significantly less LPS. We account for the possibility that pore formation can be reversed, such as in the case of transient pores that have been considered previously [1]. Pore reversal is described here as a

process that scales with pore creation, which is proportional to the concentration of AMPs and histones in the vicinity of the membrane, and occurs at a rate that is significantly lower than the rate of pore formation. Pore formation in the OM involves AMP and histone activity towards both LPS and non-LPS surfaces, which is described by the expression:

$$K_{\mathrm{OM}} = \underbrace{k_{\mathrm{al}}[A_{\mathrm{ex}}] + k_{\mathrm{hl}}[H_{\mathrm{ex}}]}_{\text{Outer leaflet OM pore formation}} + \underbrace{k_{\mathrm{anl}}[A_{\mathrm{p}}] + k_{\mathrm{hnl}}[H_{\mathrm{p}}]}_{\text{Inner leaflet OM pore formation}} - \underbrace{k_{\mathrm{r}}([A_{\mathrm{ex}}] + [H_{\mathrm{ex}}] + [A_{\mathrm{p}}] + [H_{\mathrm{p}}])}_{\text{Pore reversal}}, \tag{7}$$

where k_{al} and k_{hl} and are the rates of pore formation by AMPs and histones, respectively, towards the LPS membrane surfaces, k_{anl} and k_{hnl} are the analogous rates towards the non-LPS membrane surfaces, and k_{r} is the rate of pore reversal. Pore formation in the IM is described by activity of AMPs and histones towards only non-LPS membranes, which is described by:

$$K_{\mathrm{IM}} = \underbrace{k_{\mathrm{anl}}[A_{\mathrm{p}}] + k_{\mathrm{hnl}}[H_{\mathrm{p}}]}_{\text{Outer leaflet IM pore formation}} + \underbrace{k_{\mathrm{anl}}[A_{\mathrm{c}}] + k_{\mathrm{hnl}}[H_{\mathrm{c}}]}_{\text{Inner leaflet IM pore formation}} - \underbrace{k_{\mathrm{r}}([A_{\mathrm{p}}] + [H_{\mathrm{p}}] + [A_{\mathrm{c}}] + [H_{\mathrm{c}}])}_{\text{Pore reversal}}. \tag{8}$$

The parameters are summarized in table S1.

**Variable**	**Description**
k_{al}	Pore formation by AMPs towards LPS membrane
k_{anl}	Pore formation by AMPs towards non-LPS membranes
k_{hl}	Pore formation by histones towards LPS membrane
k_{hnl}	Pore formation by histones towards non-LPS membranes
k_{r}	Pore reversal / closing
K_{OM}	Rate of AMP or histone translation across the outer membrane
K_{IM}	Rate of AMP or histone translation across the inner membrane

Table S1: Summary of kinetic parameters.

Synergy score

An antimicrobial synergy score was computed based on the concentration of AMPs and histones in the cytoplasm. These concentrations were computed for the conditions in which cells are treated with both molecules combined or individually. The synergy score is defined as the ratio of the combined treatment to the individual treatment:

$$S = \frac{[A_{\mathrm{c}}]_{\mathrm{combined}} + [H_{\mathrm{c}}]_{\mathrm{combined}}}{[A_{\mathrm{c}}]_{\mathrm{individual}} + [H_{\mathrm{c}}]_{\mathrm{individual}}}, \tag{9}$$

where individual and combined refer to the AMPs or histones that are supplied individually or in combination as treatments, respectively. Scores of less than 1 indicate antagonism, greater than 1 indicate synergy, and a score of 1 indicates the lack of synergy or antagonism.

Simulation parameters

The simulations were performed so that synergy could be assessed using ratios of pore formation rates, which are dimensionless. For the figure on antimicrobial synergy scores for a range of histone pore formation rates against LPS and non-LPS membranes (**Fig. 5e in main text**), the pore formation rate of AMPs for both the LPS and non-LPS membranes was set equal to each other, the pore reversal rate was set to 1/10th of this value, and cooperativity was set to zero (Table S2). This corresponds to a scenario where AMPs have activity towards LPS and non-LPS surfaces, which has been observed previously [2]. The initial conditions were set such that the concentration of histones, AMPs, or both was a value of 10 in the extracellular space and zero elsewhere and the total simulation time was set to 1 s, which corresponds to a scenario in which the concentrations of AMPs and histones in all locations (extracellular space, periplasm, and cytoplasm) reach a steady state value.

**Variable**	**Value**
k_{al}	$0.01\ \mathrm{s}^{-1}$
k_{anl}	k_{al}
k_{r}	$0.1 * k_{\mathrm{al}}$

Table S2: Parameters utilized for the simulation on the range of histone pore formation rates against LPS and non-LPS membranes (**Fig. 5e in main text**).

This simulation determined synergy for a range of histone pore formation rates towards non-LPS and LPS membranes and identified maximum synergy at approximately $k_{\mathrm{hnl}} = 0.2\,\mathrm{s}^{-1}$ and $k_{\mathrm{hl}} = 0.001\,\mathrm{s}^{-1}$ (**Fig. 5e in main text**). To further understand the conditions contributing to synergy, we assessed the impact of altering the AMP pore formation rate towards non-LPS and LPS membranes in the vicinity of this histone pore formation rate ($k_{\mathrm{hl}} = 0.002\,\mathrm{s}^{-1}$). We found that synergy increased when AMP activity towards non-LPS membranes decreased and AMP activity towards LPS membranes increased (**Fig. S8 in Supplemental Information**). Together, these results show that synergy between histones and AMPs is increased when each has specific pore formation activity towards a single membrane.

Supplemental References

[1] M. Bischofberger, M. R. Gonzalez, F. G. van der Goot, Membrane injury by pore-forming proteins, Current opinion in cell biology 21 (4) (2009) 589—595. https://doi.org/10.1016/j.ceb.2009.04.003.

[2] E. Sancho-Vaello, D. Gil-Carton, P. François, E.-J. Bonetti, M. Kreir, K. R. Pothula, U. Kleinekathöfer, K. Zeth, The structure of the antimicrobial human cathelicidin LL-37 shows oligomerization and channel formation in the presence of membrane mimics, Sci. Rep. 10 (1) (2020) 17356. https://doi.org/10.1038/s41598-020-74401-5.
